# Small extracellular vesicle-encapsulated miR-181b-5p, miR-222-3p and let-7a-5p: Next generation plasma biopsy-based diagnostic biomarkers for inflammatory breast cancer

**DOI:** 10.1371/journal.pone.0250642

**Published:** 2021-04-26

**Authors:** Sarah Hamdy Ahmed, Nancy A. Espinoza-Sánchez, Ahmed El-Damen, Sarah Atef Fahim, Mohamed A. Badawy, Burkhard Greve, Mohamed El-Shinawi, Martin Götte, Sherif Abdelaziz Ibrahim

**Affiliations:** 1 Biotechnology/Biomolecular Chemistry Program, Chemistry Department, Faculty of Science, Cairo University, Giza, Egypt; 2 Department of Gynecology and Obstetrics, Münster University Hospital, Münster, Germany; 3 Department of Radiotherapy–Radiooncology, University Hospital Münster, Münster, Germany; 4 Department of Zoology, Faculty of Science, Cairo University, Giza, Egypt; 5 Biochemistry Program, Chemistry Department, Faculty of Science, Cairo University, Giza, Egypt; 6 Chemistry Department, Faculty of Science, Cairo University, Giza, Egypt; 7 Galala University, Suez, Egypt; 8 Department of General Surgery, Faculty of Medicine, Ain Shams University, Cairo, Egypt; The Ohio State University, UNITED STATES

## Abstract

Inflammatory breast cancer (IBC) is a rare, but aggressive entity of breast carcinoma with rapid dermal lymphatic invasion in young females. It is either poorly or misdiagnosed as mastitis because of the absence of a distinct lump. Small extracellular vesicles (sEVs) circulating in liquid biopsies are a novel class of minimally invasive diagnostic alternative to invasive tissue biopsies. They modulate cancer progression via shuttling their encapsulated cargo including microRNAs (miRNAs) into recipient cells to either trigger signaling or induce malignant transformation of targeted cells. Plasma sEVs < 200 nm were isolated using a modified cost-effective polyethylene glycol (PEG)-based precipitation method and compared to standard methods, namely ultracentrifugation and a commercial kit, where the successful isolation was verified by different approaches. We evaluated the expression levels of selected sEV-derived miR-181b-5p, miR-222-3p and let-7a-5p using quantitative real PCR (qPCR). Relative to non-IBC, our qPCR data showed that sEV-derived miR-181b-5p and miR-222-3p were significantly upregulated, whereas let-7a-5p was downregulated in IBC patients. Interestingly, receiver operating characteristic (ROC) curves analysis revealed that diagnostic accuracy of let-7a-5p alone was the highest for IBC with an area under curve (AUC) value of 0.9188, and when combined with miR-222-3p the AUC was improved to 0.973. Further, 38 hub genes were identified using bioinformatics analysis. Together, circulating sEV-derived miR-181b-5p, miR-222-3p and let-7a-5p serve as promising non-invasive diagnostic biomarkers for IBC.

## Introduction

Inflammatory breast cancer (IBC) is the most lethal locally advanced breast cancer variant [1]. It is a relatively infrequent aggressive form of cancer that grows within the dermal lymphatics of breast tissue. It accounts for 1 to 6% of breast cancer in western countries, while its incidence is as high as 10% within the middle East [2, 3]. Based on clinical statements, one-third of the affected breast is commonly associated with erythema, edema (orange-peel appearance) and warmness [2]. The 5- year overall survival rate of IBC is 55% shorter than that of non-inflammatory breast cancer (non-IBC), with a median survival of 2.9 to 3.8 years[4, 5]. Further, approximately 75% of IBC patients are pathologically diagnosed with lymph vascular tumor emboli, which accounts for strong metastatic behavior and poor prognosis [1]. IBC diagnosis is challenged by overlapping signs and symptoms of other diseases, and approximately 30% of newly diagnosed cases with distant metastases [6]. IBC rarity, aggressiveness and misdiagnosis resulted in delayed definition of an effective treatment [3]. The substantial pain and discomfort during collecting tissue biopsies at different time points, renders early noninvasive detection an urgent priority for better management of IBC patient’s outcome.

Liquid biopsies are emerging as a fascinating minimally invasive alternative to gold standard sampling procedures. They permit easy sequential sampling for monitoring cancer recurrence, therapy response, or any tumor genomic changes in real time, thus saving costs, time and avoiding pain associated with invasive biopsies [7]. Eukaryotic cells release complex vesicular entities that were deemed to be junk cell debris for prolonged time. It wasn’t until recent, that these were revealed to be tailor-made cell specific extracellular vesicles (EVs) [8]. The EVs circulating within liquid biopsies involve small (30–200 nm) and large (up to 1000 nm) bodies that are commonly classified according to their size and origin [9]. These nanovesicles promote and maintain cancer progression, cell-cell signaling, carcinogenesis, tumor microenvironment modulation and angiogenesis via shuttling their cargo into recipient cells [9]. EVs contain microRNAs (miRNAs), small posttranscriptional regulatory non-coding RNAs, which cleave either their target mRNA or suppress translation. miRNAs are widely used as reliable biomarkers; where their differential expression profile is accompanied by different pathological conditions [10]. Of note, free plasma circulating miRNAs were identified as biomarker candidates for breast cancer, however they were challenged by the enormous variations among clinical studies in respect to their selection and specificity to breast cancer. On the contrary, EVs-derived miRNAs are a more appealing choice for diagnostic and prognostic biomarkers and robust alternative for invasive tissue biopsies [11].

Several challenges for a quick and efficient EV isolation method are frequently reported [12]. Although ultracentrifugation is the most adopted isolation method in different studies, it is not applicable for clinical studies, where only limited volumes of samples are available. Further, laborious and extended time is needed as EV sedimentation efficiency decreases in biological fluids [13]. Additionally, repeated centrifugation cycles at higher speeds results in low sEVs yields compared to polyethylene glycol (PEG)-based isolation methods [13]. Commercially available kits are not only overpriced, but also co-isolate various larger EVs [14]. Conversely, the cost-effective PEG could be adapted in different approaches to isolate functional sEVs from liquid biopsies. Different studies have demonstrated that simple adaption of virus enrichment methods could be efficient and reproducible in enriching various EVs [15–17]. In particular, PEG of different molecular weights is a non-toxic and far less expensive precipitant for EVs than commercial polymer-based kits [18].

Hence, in the present study, we isolated sEVs through a modified PEG-based method, analyzed the expression pattern of five selected miRNAs as sEV cargo and assessed their use as alternative minimally invasive diagnostic biomarker candidates for IBC.

## Materials and methods

### Human subjects and study design

All liquid biopsy collection and preparation procedures were approved by the Institutional Review Board (IRB0006379) of Ain Shams University, Cairo, Egypt. Following the Declaration of Helsinki, all participants signed an informed consent to be enrolled in this investigation. Patients included were neither pregnant nor issued with bloodborne or autoimmune disease, while healthy subjects had no oncologic history. This prospective case-control study included 77 women; 57 were diagnosed with breast cancer (34 non-IBC and 23 IBC), while 20 were healthy volunteers used as control group. This study followed two consecutive phases: procedure optimization (n = 12) and biomarker screening phase (n = 77). Peripheral blood was collected on the same day of the curative surgery in EDTA coated vacutainers (BD Bioscience, CA, USA) and centrifuged for 10 min at 1,500 g to isolate plasma. All clinicopathological data including age, tumor size, tumor grade, lymphovascular invasion, lymph node metastasis, estrogen receptor (ER) status, progesterone receptor (PR) status, and human epidermal growth receptor 2 (HER-2) were collected from the clinical and pathological records.

### sEVs enrichment

**Fig 1:**
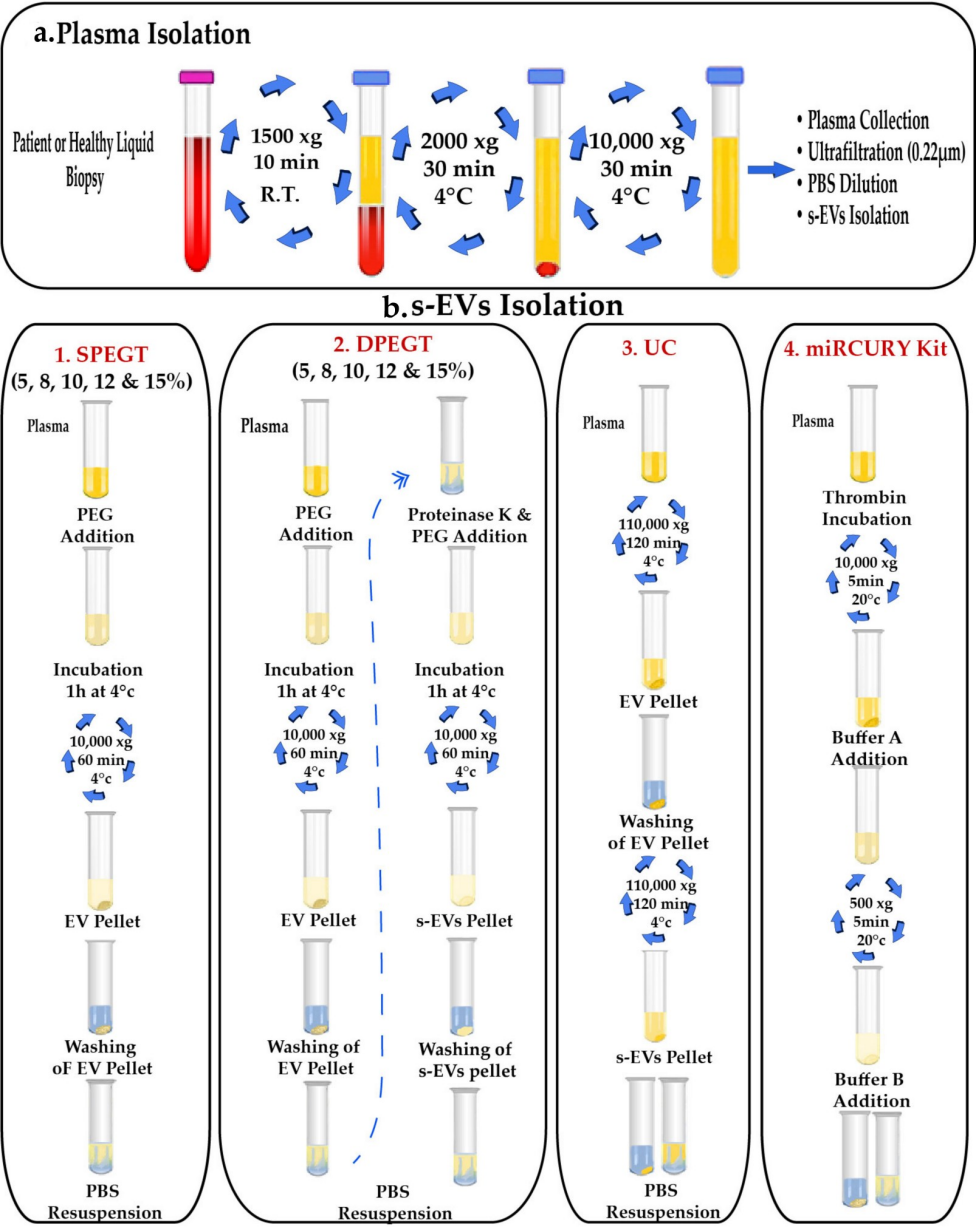
Schematic illustration of isolation procedures for sEVs. (a) Blood processing and plasma isolation. (b) Detailed steps of sEVs isolation using single PEG6000 treatment (SPEGT), double PEG6000 treatment (DPEGT), ultracentrifugation (UC) and miRCURY kit. The PEG incubation period could optionally range from 1 to 24 hrs.

For sEVs isolation procedure optimization phase, twelve plasma samples were pooled (0.25 μl / sample) and differentially centrifuged twice; at 2000 g for 10 min and 10,000 g for 30 min to eliminate cellular debris and non-EV bodies, respectively, as depicted in Fig 1A. Pooled samples were subsequently ultra-filtrated with 0.22μm syringe filters, diluted with PBS to minimize plasma viscosity, which may lead to density gradients formation that may hinder subsequent EVs pelleting, and further processing through different approaches, including single polyethylene glycol treatment (SPEGT), double polyethylene glycol treatment (DPEGT), ultracentrifugation and the miRCURY Exosome Plasma Kit (Fig 1B). Finally, EVs were either processed directly for miRNA and protein extraction or kept at -80°C prior to analysis.

### Single Polyethylene Glycol Treatment (SPEGT)

Firstly, polyethylene glycol (PEG) precipitation involved dissolving 20 grams of PEG6000 (Sigma, Cat#81260, MO, USA) in 50 mL nuclease-free water (Qiagen, Cat#129115, Hilden, Germany) to prepare 40% (w/v) sterile PEG6000 stock. This homogenous liquid stock was afterward ultra-filtrated with a 0.22μm syringe filter to exclude any contaminating particles, diluted to reach the final 5, 8, 10, 12, and 15% concentrations, and eventually added to differentially centrifuged clinical plasma pooled samples. In SPEGT, plasma pooled samples (n = 12) were mixed with the different PEG concentrations at 4°C overnight. Afterward, samples were centrifuged at 10,000 g for 1h. Then, the supernatant was aspirated and the sEVs pellet was washed with 1X PBS (Fig 1**B**1).

### Double Polyethylene Glycol Treatment (DPEGT)

The same previous liquid stock preparation and steps were followed and the initial 10k EV pellet was re-washed with 1X PBS. Additionally, 0.25 μg/μl Proteinase K (Thermo Scientific, Cat#EO0491, MA, USA) was added for 20 min at 37°C to reduce the extensive loads of circulating EVs masking proteins (e.g., albumin and lipoproteins) and followed by reprecipitating the sEV pellet using different PEG concentrations (Fig 1B2).

### Ultracentrifugation (UC)

The UC procedure was optimized following the protocol of Kim *et al*. [19], with some modifications. Pooled plasma biopsies (n = 6) were centrifuged as mentioned before, diluted with 1X PBS, and carefully transferred to ultra-clear centrifuge tubes (Thermo Scientific). EVs were precipitated at 110,000 g for 2h at 4°C using Sorval MTX 150 micro-ultracentrifuge (Thermo Scientific, S50-A 2238 rotor). The initial 110k EV pellets were subsequently washed with 1X PBS, filtered with a 0.22μm filter (Millipore), and re-centrifuged at 110,000 g for 2h at 4ᴼC. Finally, the final 110k sEV pellet was resuspended in 250 μL PBS (Fig 1B3).

### MiRCURY exosome plasma kit

Pooled plasma (n = 12) was processed according to the manufacturer’s instructions of the miRCURY Exosome Plasma Kit (Qiagen, Cat#76603). In brief, 6 μL of 500 U/mL Thrombin was added to 0.6 mL liquid biopsy, incubated for 5 min at 25°C, and centrifuged at 10,000 g for 5 min. The supernatant was gently mixed with the precipitation buffer A, incubated overnight at 4°C, and the resuspension buffer B was added to the 0.5k EV pellet (Fig 1B4).

### sEVs characterization

According to the International Society of Extracellular Vesicles (ISEV) guidelines, at least three different techniques should be used to characterize EVs [20]. Therefore, dynamic light scattering (DLS), transmission electron microscopy (TEM) and Western blot analysis were used in this study.

### Dynamic Light Scattering (DLS)

sEVs (10 μL) from the four procedures were diluted in 1 mL sterile MilliQ pure water or PBS and mixed thoroughly to attain an equally distributed homogenous solution. The size and Poly Dispersity Index (PDI) of sEVs were measured via Malvern Zetasizer Nano ZS90 (ZEN 3600, Malvern Instruments, Worcestershire, UK) (available at Cairo University Research Park, Faculty of Agriculture) equipped with a 633-nm He-Ne laser. Data were analyzed using Malvern zeta sizer software version 7.12 at a position of 4.65 mm from the cuvette wall with an automatic attenuator at a controlled temperature of 25°C. Each sample was subjected to 15 runs of 10 s and three replicates. The adapted material refractive index (RI) was 1.37 while dispersant RI was 1.33 for water and 1.34 for PBS, the viscosity was 0.89 cP for water and 1.02 cP for PBS [21].

### Transmission Electron Microscopy (TEM)

The size and morphology of sEVs were resolved by TEM. Briefly, 20–50 μL drop of the suspension was loaded onto a 200-mesh copper-coated grid for 15–30 min in a dry environment, fixed with 2% glutaraldehyde for 15 min, and the grids were rinsed four times in 100 μL MilliQ water. sEVs were stained for 10 min via a 30 μL drop of 2.5% uranyl acetate and excess liquid was removed using filter paper. Imaging was performed at 120 kV with JEOL-JEM 1200EX II Transmission Electron Microscope (Tokyo, Japan) (available at Cairo University Research Park, Faculty of Agriculture). Images were captured using a 4k*4k image resolution Eagle CCD camera.

### Protein quantification

The protein concentration of the isolated fractions was quantified using a BCA protein assay kit (Pierce, Thermo Scientific, Cat#23227) according to the manufacturer’s protocol. Briefly, bovine serum albumin (BSA) standards and samples were pipetted (25 μL/well) into the 96-microplate and mixed thoroughly with 200 μL of working reagent on a plate shaker for 30 seconds. After incubation at 37°C for 30 min, the absorbance was measured at 562 nm using Nanoquant Tecan Infinite PRO 200 (Tecan, Switzerland), and BSA standard curve was used to determine the sample protein concentration.

### Western blot

Equal sample volumes (16 μL) were lysed with 4 μL of 5X reducing sample buffer (1 M Tris-HCl (pH 6.8), 30% glycerol, 6% SDS, 3% 2-mercaptoethanol, 0.005% bromophenol blue) and boiled at 95°C for 5 min. Pooled samples (n = 12) were separated onto a 10% SDS-PAGE and electro-transferred onto a nitrocellulose membrane (Amersham, UK). Membranes were blocked with 5% non-fat skimmed milk in TBST, followed by overnight incubation with the following primary exosomes specific antibodies diluted at 1:500 HSP70 (sc-24, Santa Cruz, TX, USA), CD63 (Sc-5275, Santa Cruz), GM130 (P-20, sc-16268, Santa Cruz), Alix (sc-53540, Santa Cruz), and GAPDH (sc-20357, Santa Cruz). Subsequently, horseradish peroxidase (HRP)-conjugated anti-mouse secondary antibody (Sigma Aldrich) diluted at 1:2500 was applied for 1h. Finally, membranes were washed thrice with TBST, and the enhanced chemiluminescent (ECL) HRP substrate (Thermo Scientific, Waltham, USA) was used for signal development. Chemiluminescence was detected using the UVP Biospectrum Imaging System and Image Acquisition and Analysis Software (Analytik Jena, Cambridge, UK).

### RNA extraction

Total RNA extraction from sEVs was performed using Qiazol lysis reagent (Qiagen, 79306) following the manufacturer’s protocol. All fractions were pre-incubated with RNase A (10 mg/ml) (Thermo Scientific, Cat#EN0531) at 37°C for 40 min to eliminate cross-contamination with free circulating RNA before RNA extraction. Briefly, 350 μL sample input was mixed with three volumes of Qiazol (Qiagen), centrifuged at 12,000 g for 10 min at 4°C to remove the fatty layer particulates, and 1.5 μL of the synthetic *Caenorhabditis elegans* cel-miR-39 (MS00019789, Qiagen) was spiked into the lysate. Afterward, 200 μL chloroform was mixed with the homogenate to separate the aqueous phase and an equal volume of isopropanol along with 0.8 μg/μL of carrier MS2 RNA (Roche Diagnostics GmbH, Mannheim, Germany) were added. RNA was washed with 70% ethanol and eluted in 20 μL RNase free water, then concentrations at 230, 260 and, 280 nm were estimated using Nanoquant Tecan Infinite PRO 200 spectrophotometer (Tecan, Switzerland). RNA samples were processed directly for cDNA synthesis, then stored at -20°C till RT-qPCR analysis, where miRNA expression levels were assessed to compare efficiency of sEVs isolation methods using DPEGT, UC, and the miRCURy kit.

### Reverse Transcription Quantitative Polymerase Chain Reaction (RT-qPCR)

For reverse transcription, the mature sEV-derived miRNAs were reverse transcribed through the miScript II RT kit (Qiagen, Cat#218161). According to the manufacturer’s protocol, 3 μL of RNA was mixed with 5X HiSpec buffer, 10X Nucleic mix, and Reverse transcriptase then the reaction was set up for 60 min at 37°C followed by 5 min at 95°C. cDNA was later diluted to final concentration of 3 ng/μL for RT- qPCR using miScript SYBR Green PCR Kit (Qiagen, Cat#218073), mixed with 5 μL Qiagen SYBR Green Master Mix, universal primer, and miRNA specific upstream primer in Step One Plus Detection System (Applied Biosystems, CA, USA). The RT-qPCR thermal profile was set to 95°C for 15 min, 40 cycles of 94°C for 15 sec, 55°C for 30 sec, and 70°C for 30 sec followed by melting curve detection according to the manufacturer’s instructions. Relative to healthy controls, fold change of miRNA expression was calculated using the 2^−ΔΔCt^ method and normalized to a combination of reference miRNAs; cel-miR-39 (exogenous control, MS00019789), miR-16-3p (endogenous control, MIMAT0000069), and RNU6B (endogenous ribonuclear RNA, 001093) as no well-established set of internal controls for EV-encapsulated miRNA expression analysis have not yet been identified [22–25]. The stability of these reference miRNAs was evaluated via RefFinder; a web-based tool that integrates four computational programs (Delta-Ct, GeNorm, Normfinder, and BestKeeper) and assigns a geometric mean value for each reference miRNA based on the overall ranking obtained from each of the four statistical algorithms [26]. Five miRNAs: miR-19a-3p (MS00003192), miR-129-5p (MS00008589), miR-181b-5p (MS00006699), miR-222-3p (MS00007609), and let-7a-5p (MS00031220), were primarily screened in breast cancer patients (n = 6), then we specifically focused on sEV miRNAs that may emerge as candidate biomarkers for IBC. All primers were purchased from Qiagen.

### Bioinformatical analysis for miRNA target genes and pathways prediction

The Kyoto Encyclopedia of Genes and Genome (KEGG) pathways associated with miR-181b-5p, miR-222-3p, and let-7a-5p were analyzed in miRPath DIANA software (version 3.0) [27] based on the TarBase database (version 7.0). For the prediction of miR-181b-5p, miR-222-3p, and let-7a-5p target genes miRbase database (miRDB) (http://mirdb.org/index.html) [28, 29] was used and all the target genes with a score > 95 were selected. Then, the online Database for Annotation, Visualization and Integrated Discovery (DAVID) software was employed to analyzed the gene ontology (GO) Function and KEGG pathways associated to the target genes of miR-181b-5p, miR-222-3p, and let-7a-5p. DAVID software uses well‐known classification systems, including GO, KEGG, and BioCarta path (https://david.ncifcrf.gov/home.jsp) [30, 31]. A false discovery rate (FDR) < 0.05 was chosen as the cut‐off criterion. Finally, the integration of the protein-protein interaction (PPI) network and the identification of significant candidate genes (Hub Genes) and pathways were performed with the online platform STRING (https://string‐db.org), that uses GO, Protein families (Pfam), and KEGG databases to predict PPI networks [32]. First, all the target genes of miR-181b-5p, miR-222-3p, and let-7a-5p were uploaded to String to obtain a single network. Then, the PPI network was exported to Cytoscape software (version 3.8.0) [33] for further network analyses. The Cytoscape plugin Molecular Complex Detection (MCODE) was used to identify the most important clusters or modules from the densely/highly interconnected regions within the network. After set the parameters by the node score cutoff = 0.2, k‐core = 2, max. depth from seed = 100, and degree cutoff = 2, we selected four most significative modules with a score > 5, which containing the hub genes. Later, the hub genes were mapped into String to perform an enrichment pathway analysis.

### Statistical analysis

Unless otherwise stated, data are expressed as mean and standard deviation (SD) or standard error mean (SEM). All data were initially tested for normality using Kolomogrov Smirnov and Shapiro-Wilk normality tests. Unpaired Student’s t-test was used to compare two normally distributed groups of data, while data of more than 2 groups were analyzed using one-way ANOVA, followed by post hoc tests. The significance between non-parametric variables was evaluated using Chi-square test or Fisher’s exact test. The receiver operating characteristic (ROC) curve was calculated based on RQ values. For combined ROC curves, logistic regression models were used to determine the predictive values. The area under the curve (AUC) with 95% CI was calculated for each ROC curve, from which the optimal cut-off point, sensitivity, and specificity of each miRNA were determined. Data were analyzed using both GraphPad Prism 8 (GraphPad Software Inc., San Diego, CA, USA) and IBM SPSS advanced statistics version 25 (SPSS Inc., Chicago, IL, USA). Data were considered significant when p-value is < 0.05.

## Results

### Characterization of plasma-derived sEVs

**Fig 2:**
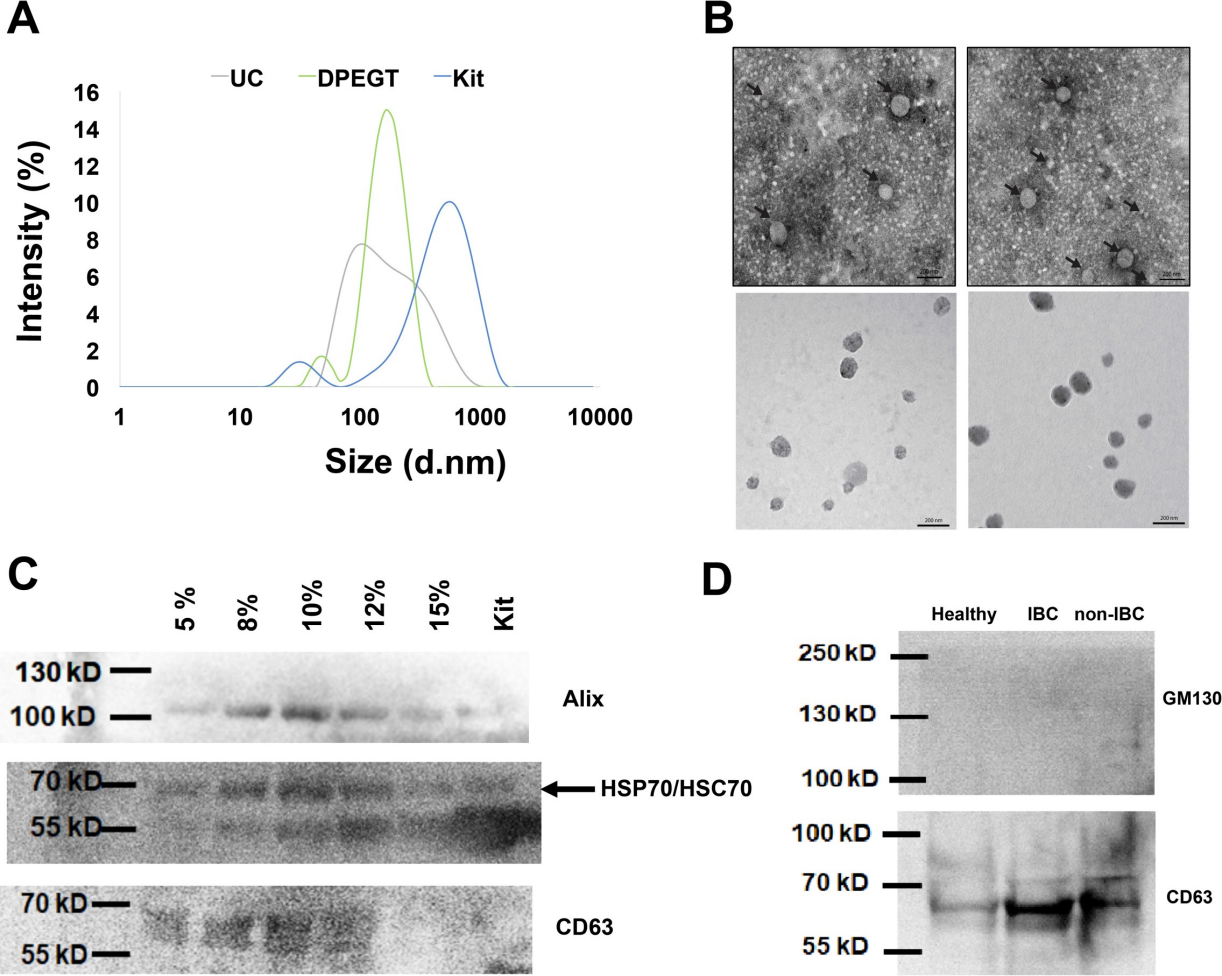
Characterization of enriched plasma sEVs of breast cancer patients. The successful isolation of plasma sEVs was verified by different methods. (a) sEVs size distribution was determined by dynamic light scattering (DLS). (b) Transmission electron micrograph of negatively stained sEVs. Size bar equal 200 nm. (c) Western blot of the exosomal proteins markers; Alix, CD63 and HSP70/HSC70 using different concentrations of DPEGT (pooled, n = 12) in comparison to the miRCURY kit. (d) Western blot analysis of the exosomal proteins (e.g., CD63) and non-exosomal Golgi apparatus protein GM130.

Different transcriptomic and proteomic validation approaches, including DLS, TEM, and western blot were conducted to ascertain the successful isolation of sEVs. First, we isolated sEVs through ultracentrifugation, combined differential centrifugation followed by PEG precipitation (i.e. SPEGT and DPEGT), and the miRCURY Exosome Plasma Kit to evaluate which method could be efficiently employed in the screening of sEV-derived miRNAs as potential biomarkers for IBC relative to non-IBC patients. sEVs heterogeneity was measured in terms of the polydispersity index (PDI), where the lower the PDI, the more monodispersed EV fractions. Based on PDI measurements, EVs with 0.377–0.569 PDI values were isolated using ultracentrifugation and the miRCURY kit, while SPEGT and DPEGT with different concentrations showed 0.558–0.616 and 0.295–0.528 PDI, respectively (Table 1). Notably, the PDI of DPEGT was significantly improved compared to that of SPEGT (*p* < 0.05), indicating that the extra enzymatic and washing steps in DEPGT (8%, PDI = 0.29) have massively enhanced the homogeneity of the retrieved EVs and minimized the polydispersed aggregates. DLS analysis was used to assess the hydrodynamic radius of sEVs and showed that DPEGT and ultracentrifugation resulted in sEVs with an average size less than 200 nm. Of note, the highest intensity was obtained with the 8% DPEGT (Fig 2A). The size distribution of sEVs isolated with different concentrations of SPEGT and DPEGT were compared to ultracentrifugation and miRCURY kit (S1 Fig). In agreement with DLS data, TEM analysis of ultracentrifugation and DPEGT methods showed small rounded or oval-shaped EVs (sEVs) (< 200 nm) with an average size ranging from 75–105 nm for both ultracentrifugation and DEPGT, respectively as depicted in Fig 2B. Western blot analysis of sEVs-enriched pooled fractions by DPEGT (n = 12) confirmed the efficacy of 8% DPEGT in enrichment of sEVs, specifically exosomes. Western blot analysis verified the presence of the Alix, CD63 and HSP70/HSC70 exosomal proteins markers (Fig 2C) using different concentrations of DPEGT compared to miRCURY kit. To further confirm the success of isolation, sEVs were isolated from three different samples namely normal pooled samples (n = 12), non-IBC pooled samples (n = 12) and IBC pooled samples (n = 12) and their western blot analysis unveiled detectable exosomal markers, namely CD63 (tetraspanin) (Fig 2D). On the contrary, no cell organelle contaminating proteins, such as non-exosomal Golgi apparatus protein (GM130) was detected (Fig 2D). We next compared the different approaches employed through the proteomic, transcriptomic yield and purity of enriched plasma sEVs.

**Table 1:** Polydispersity index (PDI) of plasma sEVs enriched by different procedures.

Procedure	UC	miRCURY Kit	SPEGT					DPEGT				
			5%	8%	10%	12%	15%	5%	8%	10%	12%	15%
**PDI**	**0.377**	**0.569**	**0.616**	**0.566**	**0.558**	**0.579**	**0.6**	**0.359**	**0.295**	**0.488**	**0.51**	**0.528**

UC: ultracentrifugation; SPEGT: single polyethylene glycol treatment; DPEGT: double polyethylene glycol treatment.

### Proteomic content

**Fig 3:**
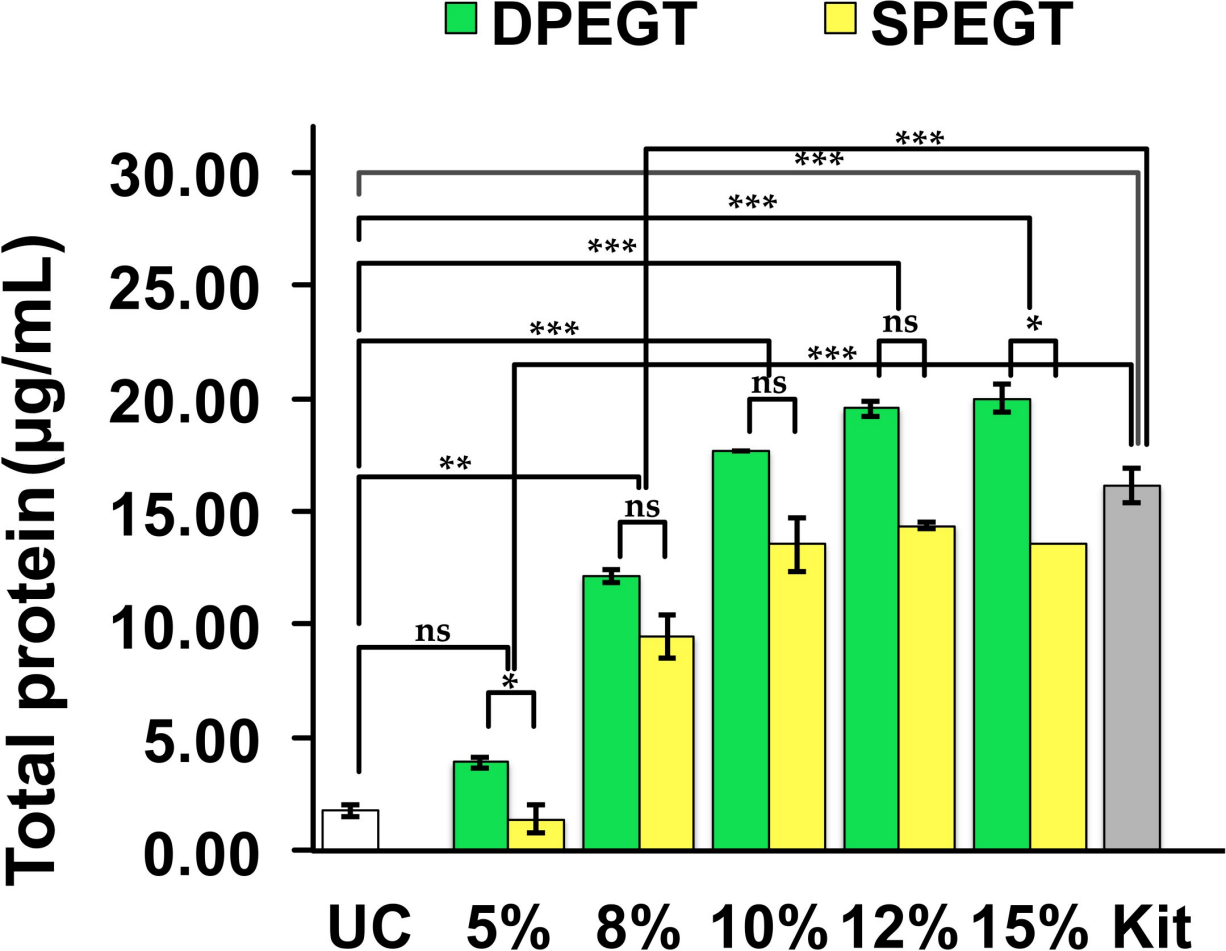
Total protein content of enriched plasma sEVs. Isolation of sEVs was conducted by PEG-based methods SPEGT, DPEGT, and the miRCURY kit and UC. Data represent the mean ± SEM, n = 12. *** p < 0.001, ** p < 0.01, * p < 0.05 and ns (not significant) as determined by either Student’s t-test or ANOVA test.

During the training phase, plasma biopsies were pooled (n = 12) to isolate sEVs using the aforementioned methods and BCA assay was primarily conducted to measure the protein content of sEVs. It was observed that protein content of sEVs increased upon increasing PEG concentration in both single and double treatments, whereby DPEGT generally yielded higher protein content than SPEGT. As depicted in Fig 3, the protein content of sEVs enriched through 5% and 15% of DPEGT were significantly higher than those of SPEGT (*p* < 0.05). Also, the protein content of sEVs had a trend of significance with concentrations of 8% (*p* = 0.08), 10% (*p* = 0.07) and 12% (*p* = 0.08) of DPEGT compared with SPEGT (Fig 3). sEVs isolated by the miRCURY kit showed significantly higher protein yield than the 5% and 8% concentrations of SPEGT and DPEGT (*p* < 0.001, for both), whereas ultracentrifugation resulted in limited protein yield compared to 8% (*p* < 0.01), 10% (*p* < 0.001), 12% (*p* < 0.001) and 15% (*p* < 0.001) concentrations of both SPEGT and DPEGT. Collectively, these results suggest that relative to ultracentrifugation, PEG-based methods (SPEGT, DPEGT and the miRCURY kit) may contribute to the enrichment of more plasma sEVs as reflected by higher protein yield.

### Transcriptomic content

**Fig 4:**
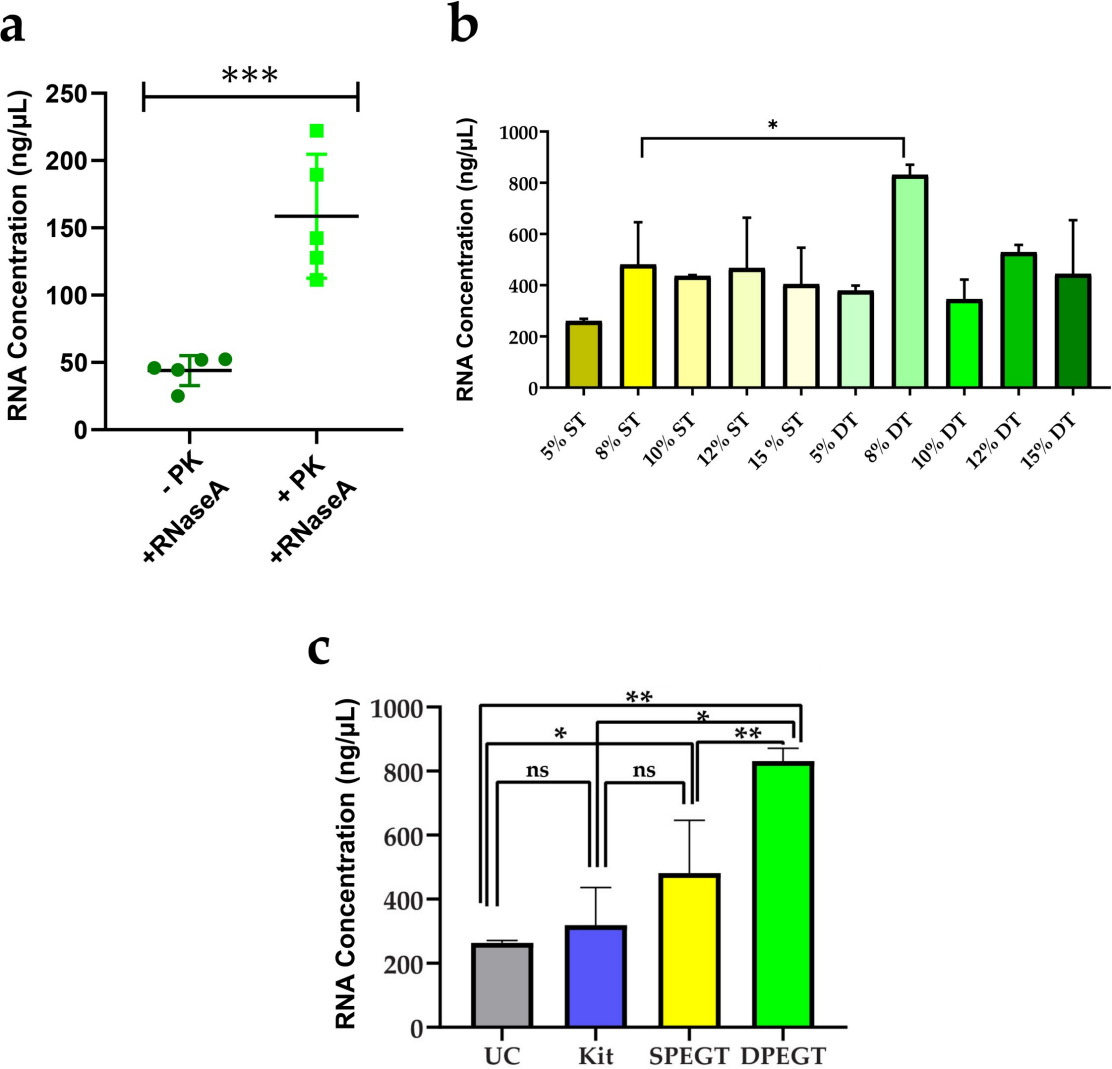
Total RNA concentration of enriched plasma sEVs. Isolation of sEVs was conducted by PEG-based methods SPEGT, DPEGT, and miRCURY kit and UC. (a) Effect of RNase A with or without proteinase k (PK) treatment on RNA yield. *** p < 0.001 as determined by Student’s t-test, n = 5. (b) Effect of single treatment (ST) and double treatment (DT) of PEG with different concentrations on total RNA yield. Data represent the mean ± SEM. * p < 0.05 as determined by Student’s t-test. (c) Total RNA yield upon 8% SPEGT, 8% DPEGT, miRCURY kit and UC. Data represent the mean ± SEM. ** p < 0.01, * p < 0.05 and ns (not significant) as determined by either Student’s t-test or ANOVA test.

As RNA quantity may reflect the total sEVs yield, total RNA of all isolated sEVs fractions was measured. First, plasma biopsies were pooled for each treatment (n = 12) and total RNA of enriched sEVs with different concentrations of SPEGT and DPEGT was compared to confirm that plasma sEVs were not masked with numerous circulating proteins and total RNA was confined to enriched sEVs, consecutive RNase A and proteinase K treatments of sEVs were used in DPEGT before total RNA extraction. Afterwards, RNA concentration was determined in pooled samples (n = 5) treated with both enzymes and samples (n = 5) treated with only RNase A. Samples treated with Proteinase K had significantly 3.6-fold higher RNA content than those without proteinase K treatment (*p* < 0.001; Fig 4A), which emphasizes that excessive high molecular weight circulating proteins can affect the isolation of sEVs encapsulated RNAs. A significant difference for total RNA yields was observed between different PEG concentrations (p < 0.05), however, total RNA isolated from sEVs by 8% DPEGT was significantly higher than that of SPEGT (*p* < 0.05, Fig 4B). Next, 8% of both SPEGT and DPEGT were compared to the miRCURY kit and ultracentrifugation. DPEGT with 8% significantly increased sEV-derived RNA content compared to other methods (*p* < 0.01; Fig 4C). Moreover, as depicted in Fig 4C, 8% DPEGT significantly increased sEV-derived RNA yield by relative to ultracentrifugation (by 4-fold; *p* < 0.01), SPEGT (*p < 0*.*01*) and the miRCURY kit (*p < 0*.*05*).

### Expression profile of plasma sEV-derived miRNAs in IBC versus non-IBC

**Fig 5:**
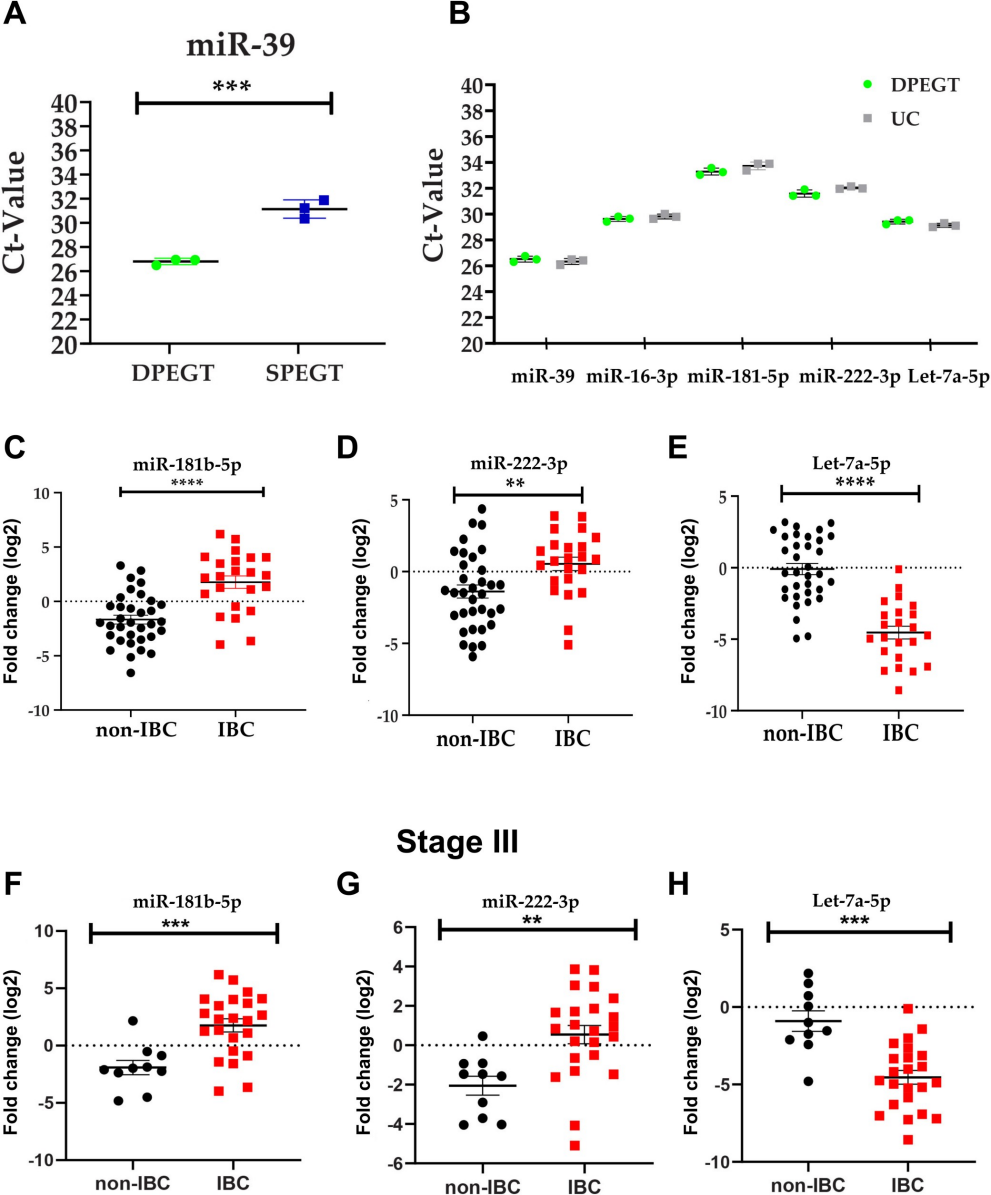
Expression levels of miR-181b-5p, miR-222-3p and let-7a-5p in enriched plasma sEVs. Total RNA of sEVs isolated from patients with non-IBC and IBC, and normal volunteers was reverse transcribed into cDNA and miRNA expression levels were quantified using specific miRNAs primers. (a) Ct values of spike in miR-39. n = 3, *** p < 0.001 as determined by Student’s t-test. (b) Comparable Ct values of miR-39, miR-16-3p, miR-181b-5p, miR-222-3p, and let-7a-5p of enriched pooled plasma sEVs isolated by DPEGT and UC, n = 3. Relative expression levels of miR-181b-5p (c), miR-222-3p (d), and let-7a-5p (e) in enriched plasma sEVs isolated from IBC (n = 23) and non-IBC (n = 34) by DPEGT. **** p < 0.0001, ** p < 0.01 as determined by Student’s t-test. Relative expression levels of miR-181-5p (f), miR-222-3p (g) and let7-5p (h) in enriched plasma sEV isolated from stage III of both IBC (n = 23) and non-IBC (n = 10) by DPEGT. *** p <0.001, ** p < 0.01 as determined by Student’s t-test.

We have recently discovered differentially expressed miRNAs in IBC tumors relative to non-IBC as tissue biomarkers, using human breast cancer miRNA PCR array [34]. To extend our findings and to identify sEV-derived miRNAs as non-invasive blood-based biomarkers for IBC, we selected five miRNAs (miR-19a, miR-129-5p, miR-181b-5p, miR-222-3p, and let-7a-5p). We analyzed the expression level of the spike in cel-miR-39 in pooled plasma sEVs (n = 3) enriched by 8% of both SPEGT and DPEGT. A statistically significant difference in the Ct value of cel-miR-39 was detected between DPEGT and SPEGT (p < 0.001, Fig 5A). Notably, no significant difference was detected in the Ct values of cel-miR-39, miR-16-5p, miR-181b-5p, miR-222-3p and let-7a-5p (*p* > 0.05 for all, Fig 5B). Therefore, we used DPEGT to enrich IBC and non-IBC plasma sEVs and quantify their differential miRNA expression pattern. Clinical-pathological features of IBC and non-IBC patients are depicted in Table 2. There was no significant difference in age, lymphovascular invasion, lymph node status, tumor size, tumor grade, or receptor status between IBC and non-IBC patients. However, a significant difference was found in tumor stage between IBC and non-IBC patients (*p* < 0.0001). We quantified the expression levels of miR-19a, miR-129-5p, miR-181b-5p, miR-222-3p, and let-7a-5p in enriched plasma of sEVs of patients with non-IBC (n = 34) and IBC (n = 23) relative to the expression of healthy volunteers (n = 20) by qPCR. To normalize the expression level of sEV-derived miRNAs, three reference/housekeeping controls (cel-miR-39, miR-16-3p, and RNU-6) were selected and their comprehensive stability was evaluated using computational ReFfinder software. According to their geometric mean, reference controls cel-miR-39, miR-16-3p, and RNU-6 were ordered and ranked as 1.19, 1.89, and 2.28 stability values, respectively (S2 Fig). Out of the five sEV-derived miRNAs, three miRNAs showed a significantly differential expression between IBC and non-IBC patients. miR-181b-5p and miR-222-3p expression levels were significantly up-regulated (> 2-fold) (Fig 5C; *p* < 0.0001 and Fig 5D; *p* < 0.01), whereas let-7a-5p was significantly downregulated (< 0.5-fold) (Fig 5E; *p* < 0.0001) in enriched plasma sEVs of IBC relative to those of non-IBC patients. In contrast, miR-19a and miR-129-5p expression levels were not significantly altered. Next, we compared expression levels of miR-181b-5p, miR-222-3p, and let-7a-5p in enriched plasma sEVs of IBC patients (n = 23) with those of stage-matched non-IBC patients (stage III, n = 10). Interestingly, similar findings were observed with significantly elevated expression levels of miR-181b-5p (*p* < 0.001, Fig 5F) and miR-222-3p (*p* < 0.01, Fig 5G), whilst expression levels of let-7a-5p were significantly downregulated (*p* < 0.001, Fig 5H) in enriched plasma EVs of IBC relative to non-IBC patients. However, when we stratified non-IBC according to tumor stage, no significant difference was observed for the expression levels of the 3-miRNAs between stage III (n = 10) vs. stage I & II (n = 24) (*p* > 0.05, S3 Fig). Finally, we compared the expression levels of the three miRNAs in non-IBC patients treated with neoadjuvant chemotherapy (n = 12) relative to untreated non-IBC (n = 19). Expression levels of only let-7a-5p were significantly diminished in treated non-IBC relative to untreated group (*p <* 0.01, S4 Fig). However, we did not detect any significant alterations in miR-181-5p or miR-222-3p expression levels between treated and untreated patients (*p* > 0.05, S4 Fig).

**Table 2:** Clinic-pathological features of non-IBC and IBC patients.

Group Characteristics		non-IBC	IBC	*p-*value
		n = 34	n = 23	
**Age (Years)**	Range	25–70	36–71	0.23 ^a^
	Mean (SEM)	47.41 ± 0.11	51.72 ± 0.15	
**Tumor Size (cm), n%**	≥4	19 (56)	14 (61)	0.60 ^b^
	< 4	15 (44)	9 (39)	
**Tumor Grade, n%**	I	3 (9)	2 (9)	0.75 ^b^
	II	24 (71)	17 (74)	
	III	7 (21)	4 (17)	
**Tumor Stage**	I and II	24 (71)	0	< 0.0001^b^
	III	10 (29)	23 (100)	
**Lymphovascular Invasion, n%**	Positive	13 (38)	12 (52)	0.20 ^b^
	Negative	21 (62)	11 (48)	
**Lymph Nodes, n%**	≥4	15 (44)	12 (52)	0.21 ^b^
	< 4	19 (56)	11 (48)	
**ER, n%**	Positive	23 (68)	13 (57)	0.41 ^b^
	Negative	11 (32)	9 (39)	
	NA	0	1 (4)	
**PR, n%**	Positive	24 (71)	12 (52)	0.18 ^b^
	Negative	10 (29)	10 (43)	
	NA	0	1 (4)	
**Her-2, n%**	Positive	10 (29)	10 (43)	0.15 ^b^
	Negative	24 (71)	12 (52)	
	NA	0	1 (4)	

Data are expressed as mean ± SEM, NA Data not available

* Significant p value calculated by ^a^Student’s t-test or ^b^Pearson Chi-Square. ER, estrogen receptor; PR, progesterone receptor; Her-2, human epidermal growth factor receptor-2.

### Correlations between the expression levels of miR-181b-5p, miR-222-3p and let-7a-5p

**Fig 6:**
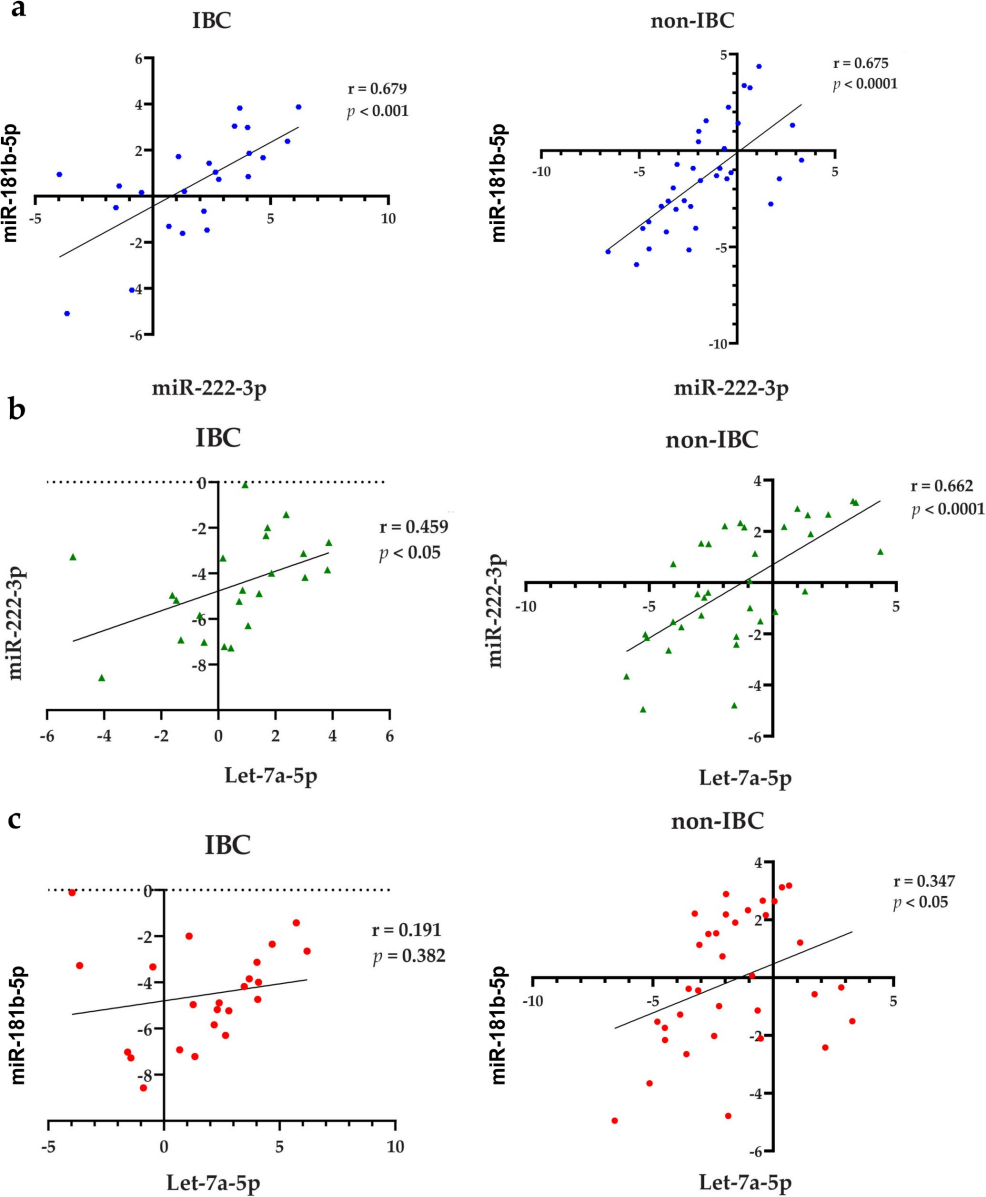
Pearson’s correlation analysis between miR-181b-5p, miR-222-3p, and let-7a-5p expression in sEVs. (a) A significant positive correlation between miR-181b-5p and miR-222-3p in IBC (left panel) and non-IBC (right panel). (b) A significant positive correlation between miR-222-3p and let-7a-5p in IBC (left panel) and non-IBC (right panel). (c) A significant positive correlation between miR-181b-5p and let-7a-5p in non-IBC (right panel), but not in IBC (left panel).

Pearson’s correlation analysis was used to determine the correlation between miR-181b-5p, miR-222-3p and let-7a-5p in IBC and non-IBC patients. A positive correlation between miR-181b-5p and miR-222-3p expression levels was detected in IBC (r = 0.679, *p* < 0.001) and non-IBC (r = 0.675, *p* < 0.001) (Fig 6A). Likewise, a positive correlation was noticed between the expression levels of miR-222-3p and let-7a-5p in IBC (r = 0.459, *p* < 0.05) and non-IBC (r = 0.662, *p* < 0.001) (Fig 6B). Although a significant correlation was observed between miR-181b-5p and let-7a-5p in non-IBC (r = 0.347, *p* < 0.05), no association was detected in IBC patients (r = 0.191, *p* > 0.05) (Fig 6C).

### Diagnostic values of sEV-derived miR-181b-5p, miR-222-3p and let-7a-5p

**Fig 7:**
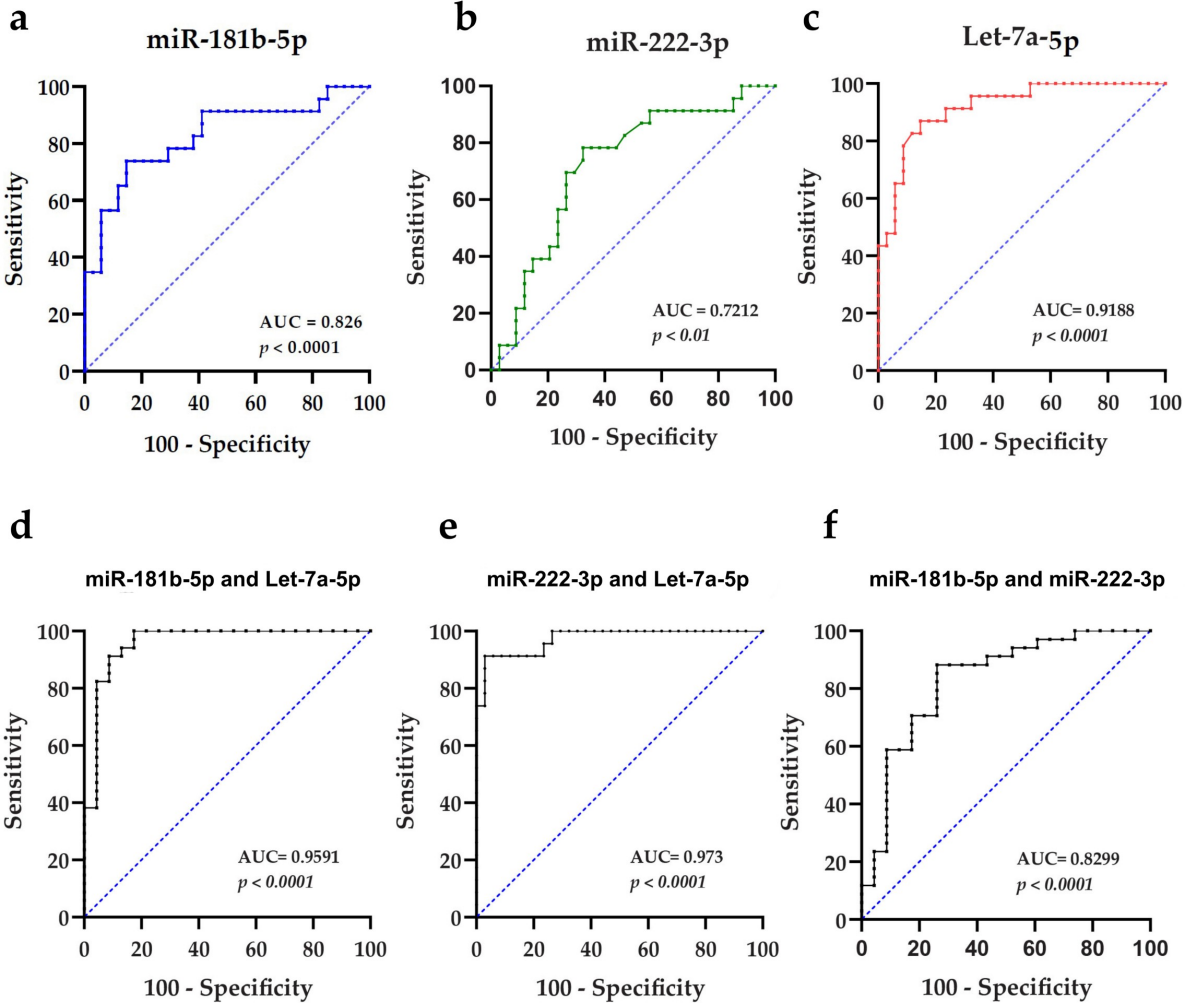
ROC curves generated using expression levels of miR-181b-5p, miR-222-3p, and let-7a-5p. (a-c) ROC curves generated using individual miRNA, namely miR-181b-5p, miR-222-3p, and let-7a-5p, respectively. (d-f) ROC curves generated using a combination of expression levels of miR-181b-5p and let-7a-5p, miR-222-3p and let-7a-5p, and miR-181b-5p and miR-222-3p as a miRNA panel to discriminate non-IBC and IBC patients, respectively.

We next assessed the diagnostic values of let-7a-5p, miR-181b-5p, and miR-222-3p by ROC curve analysis to discriminate IBC from non-IBC patients. ROC curves revealed their potential as candidate non-invasive sEV-encapsulated biomarkers, where let-7a-5p was the best individual diagnostic biomarker with an AUC of 0.9188 and optimal cutoff value of -2.255 (87% sensitivity and 85.3% specificity; *p* < 0.001) (Fig 7A), miR-181b-5p showed an AUC of 0.826 (73.9% sensitivity and 85.3% specificity; *p* < 0.001) (Fig 7B), and miR-222-3p had an AUC of 0.7212 (78.3% sensitivity and a 67.6% specificity; *p* < 0.01) (Fig 7C). Further improvement in the discriminatory accuracy of these miRNAs was expected by combination. Thus, we applied different binary logistic regression models with non-IBC as a reference category; each couple of miRNAs was combined and the positive predictive value (PPV), negative predictive value (NPV), sensitivity, specificity, and optimal cutoff values for each model were evaluated (Table 3). Using the logistic regression model, we assessed the predictive potential of different combinations of let-7a-5p, miR-181b-5p, and miR-222-3p expression levels for IBC (Table 4). The best regression model with highest accuracy was the combination of miR-222-3p and let-7a-5p expression, it was statistically significant, χ2(2) = 55.19 (p < 0.001), as it correctly classified 94.0% of cases and explained 83.0% (Nagelkerke R2) of the variance in IBC. As depicted in Table 4, the regression coefficient for let-7a-5p significantly [B = -1.21, OR = 0.30 (*p* < .001)] showed that upregulated let-7a-5p expression levels were only 0.3 times associated with the likelihood of exhibiting IBC (*p* < 0.01); in other words, downregulated let-7a-5p was significantly associated with the likelihood of exhibiting IBC. While the regression coefficient for miR-222-3p significantly [B = 0.81, OR = 2.25 (p = 0.003)] indicated that the upregulated miR-222-3p expression levels were 2.25 times more in IBC than non-IBC (p < 0.05). Hence, the ROC curve analysis of miR-222-3p and let-7a-5p panel significantly improved the AUC to 0.973 with the highest sensitivity (91.3%) and specificity (97.1%) values (p < 0.001) (Fig 7D). Of note, let-7a-5p and miR-181b-5p panel also presented an improved an AUC of 0.959 with 82.6% sensitivity and 100% specificity (p < 0.001) (Fig 7E). A combination of miR-181b-5p and miR-222-3p showed a 0.83 AUC value with a sensitivity of 73.9% and 88.2% specificity (Fig 7F). Taken together, these results suggest that sEV-derived let-7a-5p, a panel of sEV-encapsulated let-7a-5p and miR-181b-5p or let-7a-5p and miR-222-3p are promising biomarkers for accurate diagnosis of IBC.

**Table 3:** ROC curve analysis of individual and combination of sEV-derived miRNAs to differentiate IBC from non-IBC patients.

EV- derived miRNAs	let-7a-5p	miR-181b-5p	miR-222-3p	miR-222-3p and let-7a-5p panel	let-7a-5p and miR-181b-5p panel	miR-181b-5p and miRNA-222-3p panel
AUC	0.919	0.826	0.721	0.973	0.959	0.830
S. E.	0.036	0.059	0.069	0.018	0.029	0.058
95% CI	0.85–0.99	0.71–0.94	0.59–0.86	0.93–1.00	0.90–1.00	0.72–0.94
Optimal Cutoff	-2.255	0.670	-0.685	0.533	0.742	0.494
SN (%)	87.0%	73.9%	78.3%	91.3%	82.6%	73.9%
SP (%)	85.3%	85.3%	67.6%	97.1%	100%	88.2%
PPV (%)	85.7%	76.2%	60.0%	95.5%	87.0%	81.0%
NPV (%)	86.1%	80.6%	70.3%	94.3%	91.2%	83.3%
*p-*value	< 0.001	< 0.001	< 0.01	< 0.001	< 0.001	< 0.001

AUC: area under the curve; CI: confidence interval; SN: sensitivity; SP: specificity; PPV: positive predictive value; and NPV: negative predictive value.

**Table 4:** Logistic regression miRNAs combination models for IBC diagnosis.

EV-derived miRNAs		*B*	*SE*	95% CI	*OR*	Percentage Accuracy in classification (PAC)	Nagelkerke R^2^ (%)	*p*-value
miR-222-3p and let-7a-5p panel	**miR-222-3p**	0.811	0.27	[0.28, 1.34]	2.25	94.7%	83.8%	< 0.01
	**let-7a-5p**	-1.211	0.35	[-1.89, -0.53]	0.30			< 0.001
Let-7a-5p and miR-181b-5p panel	**let-7a-5p**	-0.915	0.29	[-1.48, -0.35]	0.40	89.5%	78.1%	< 0.001
	**miR-181b-5p**	0.442	0.15	[0.15, 0.74]	1.56			< 0.001
miR-181b-5p and miRNA-222-3p panel	**miR-181b-5p**	0.543	0.17	[0.21, 0.88]	1.72	82.5	41.2%	< 0.001
	**miRNA-222-3p**	-0.061	0.17	[-0.40, 0.28]	0.94			0.724

B: Beta regression coefficient; SE: Standard Error; 95% CI: confidence interval; OR: Odds Ratio.

### Prediction of miR-181b-5p, miR-222-3p and let-7a-5p target genes, GO function and KEGG pathway analysis

To identify the potential target genes of miR-181b-5p, miR-222-3p, and let-7a-5p we used the miRbase database (miRDB). After selecting all target genes of score > 95, we found 204 target genes for the three miRNAs. Then, to analyze the functional and network pathways of the selected 204 miRNAs targets, we employed the DAVID tool. The GO analysis showed the biological process (BP), cellular component (CC), and the molecular function (MF) related to all the miRNAs target genes. Table 5 shows the most eight significant terms (*p* < 0.05) in each category. In the BP category, the regulation of transcription, somatic stem cell population maintenance, mRNA destabilization and insulin growth factor receptor signaling pathway were mainly enriched. The CC category was enriched in the terms of nucleus, intracellular, endosome, heterochromatin, cytoplasmic vesicle membrane, and histone methyl transferase, glycerol-phosphate dehydrogenase, and heterotrimeric G-protein complex. Finally, DNA, metal ion, SMAD, nucleic acid, methylated histone, and transcriptional repressor activity, RNA polymerase II core promoter proximal region sequence-specific binding were enriched in the MF. Finally, the KEGG analysis demonstrated that different pathways that have been associated to cancer, including signaling pathways related to stem cells, FoxO, GABAergic and cholinergic synapse, PI3K-AKT, and Rap1 were mainly enriched (Table 6).

**Table 5:** GO analysis of miR-181b-5p, miR-222-3p and let-7a-5p target genes.

Category	Term	Count	%	p-value
**Biological process**	Regulation of transcription, DNA-templated	37	18.9	2.60E-06
	Transcription, DNA-templated	41	20.9	2.90E-05
	Anterior/posterior pattern specification	6	3.1	1.60E-03
	Positive regulation of transcription from RNA polymerase II promoter	22	11.2	1.70E-03
	Negative regulation of transcription from RNA polymerase II promoter	18	9.2	1.70E-03
	Somatic stem cell population maintenance	5	2.6	5.00E-03
	3’-UTR-mediated mRNA destabilization	3	1.5	6.90E-03
	Insulin-like growth factor receptor signaling pathway	3	1.5	9.40E-03
**Cellular component**	Nucleus	83	42.3	4.30E-05
	Intracellular	27	13.8	1.30E-03
	Endosome	9	4.6	2.40E-03
	Heterochromatin	3	1.5	2.00E-02
	Histone methyltransferase complex	3	1.5	2.30E-02
	Glycerol-3-phosphate dehydrogenase complex	2	1	3.10E-02
	Cytoplasmic vesicle membrane	5	2.6	4.20E-02
	Heterotrimeric G-protein complex	3	1.5	4.60E-02
**Molecular function**	DNA binding	39	19.9	4.10E-06
	Metal ion binding	43	21.9	1.90E-05
	Transcription factor activity, sequence-specific DNA binding	23	11.7	5.10E-04
	SMAD binding	5	2.6	1.10E-03
	Nucleic acid binding	22	11.2	1.70E-03
	Methylated histone binding	4	2	1.80E-02
	Transcriptional repressor activity, RNA polymerase II core promoter proximal region sequence-specific binding	5	2.6	3.00E-02
	Zinc ion binding	20	10.2	4.00E-02

**Table 6:** Kyoto Encyclopedia of Genes and Genomes (KEGG) enrichment pathway analysis of miR-181b-5p, miR-222-3p and let-7a-5p target genes.

Term	Count	%	*p*-value
Signaling pathways regulating pluripotency of stem cells	7	3.6	1.10E-03
FoxO signaling pathway	6	3.1	5.20E-03
GABAergic synapse	5	2.6	5.50E-03
PI3K-Akt signaling pathway	9	4.6	7.90E-03
Rap1 signaling pathway	7	3.6	8.10E-03
MicroRNAs in cancer	8	4.1	9.70E-03
Melanogenesis	5	2.6	9.80E-03
Cholinergic Synapse	5	2.6	1.40E-02
Pathways in cancer	9	4.6	1.60E-02
Morphine addiction	4	2	4.10E-02

### Identification of hub genes and enrichment pathways from miR-181b-5p, miR-222-3p and let-7a-5p targets PPI networks

**Fig 8:**
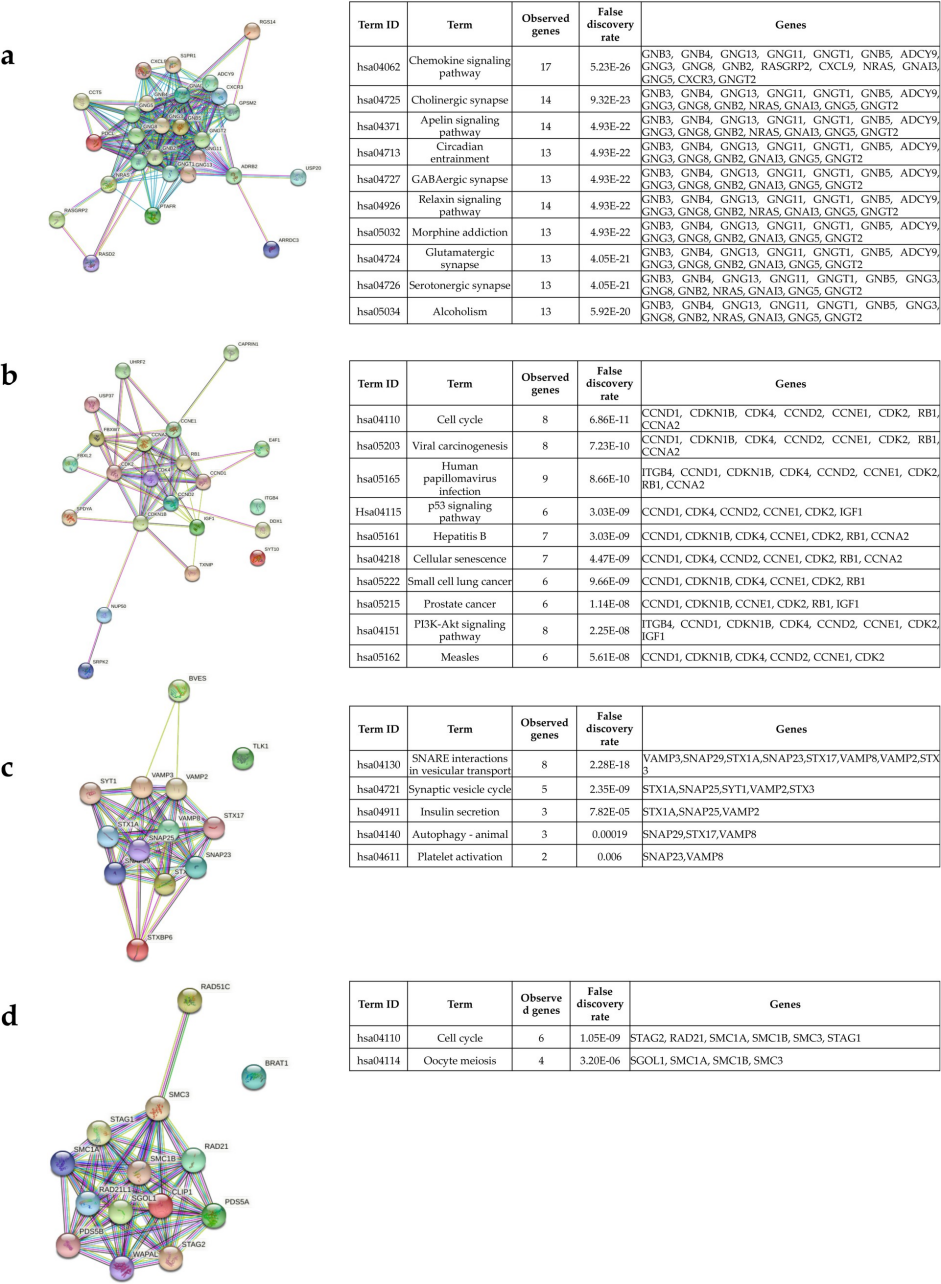
Hub genes protein‐protein interactions (PPI) and enrichment pathways. To analyze the hub genes, present in most significant modules obtained from the Cytoscape plugin Molecular Complex Detection (MCODE) we used the String database. (a-d) shows the PPI network of the hub genes present in each module and the enrichment pathways associated to them. < 1.0E‐16 is the *p*‐value for the entire PPI network of each module. String performs the false discovery rate (FDR) for the statistics.

In order to identify the hub genes, we first obtained the PPI regulatory network of the all 204 miRNAs targets. For this purpose, we used String database selected a medium score > 0.4 to construct the network. We observed a huge network in which almost all the genes were interconnected (S5 Fig). After that, the network was imported to the Cytoscape tool to obtain the hub genes. The MCODE plug in Cytoscape was used to find the modules in which the hub genes were present. S1 Table shows the 10 clusters or modules obtained after the analysis. The top four significative modules with a score > 5 in which the hub genes are present were selected. Of a total of 204 genes analyzed, 38 are hub genes. In the first module there are 19 genes, in the second module 9 and in the third and fourth module 5 genes each (S1 Table). It is very important to explore the pathways that are potentially regulated by these hub genes. To this end, the genes of each module were uploaded to the String database to obtain the PPI network and the enriched KEGG pathways of each module. At first, it was noted that all the genes of each module were highly interconnected (Fig 8A–8D). The top 10 significant (*p* < 0.05) enrichments for module 1 and 2, while only five enrichments for module 3, and two enrichments for module 4 were selected to be represented in the tables in Fig 8A–8D. The hub genes in module 1 were associated with the regulation of chemokine, apelin, and relaxin signaling pathways, cholinergic, GABAergic, glutamatergic, and serotonergic synapse, circadian, morphine addiction, and alcoholism (Fig 8A). Interestingly, the hub genes in module 2 potentially regulate pathways associated to cancer such as p53 and PI3K-AKT signaling, cell cycle, cellular senescence, small cell lung and prostate cancer, and viral carcinogenesis (Fig 8B). On the other hand, the hub genes of module 3 have an impact on vesicular transport, synaptic vesicle circle, insulin secretion, autophagy, and platelet activation (Fig 8C). Finally, hub genes in module 4 were more associated with cell cycle and meiosis (Fig 8D).

### Pathways associated with miR-181b-5p, miR-222-3p and let-7a-5p expression

We used the DIANA miRPath online tool to obtain the KEGG enrichment pathways of miR-181b-5p, miR-222-3p, and let-7a-5p. We found 26 pathways with significant *p*-values, there were common pathways that all three miRNAs share including p53 signaling and ubiquitin mediated proteolysis (Table 7 and S6 Fig). On the other hand, let-7a-5p was associated with pathways related to cancer, such as TGF-β, thyroid hormone signaling, transcriptional mis-regulation, other pathways in cancer, colorectal carcinoma, chronic myeloid leukemia, Hepatitis B, endocytosis and viral carcinogenesis (Table 7 and S6 Fig). miR-222-3p and let-7a-5p shared the enrichment of HTLV-infection, arrhythmogenic cardiomyopathy, lysine degradation, and cell cycle signaling. The enrichment of Hippo signaling and proteoglycans in cancer were associated with miR-181b-5p and let-7a-5p. miR-181b-5p has an important regulatory role in prostate and renal cell cancer, whilst miR-222-3p regulates cell adhesion molecules (Table 7 and S6 Fig).

**Table 7:** Kyoto Encyclopedia of Genes and Genomes (KEGG) pathways associated with miR-181b-5p, miR-222-3p, and let-7a-5p.

KEGG pathway	Associated miRNAs	Number of genes	*p*-value
Lysine degradation	hsa-miR-222-3p	17	1.25E-09
	hsa-let-7a-5p		
Hippo signaling pathway	hsa-let-7a-5p	52	4.10E-09
	has-miR-181b-5p		
Cell cycle	hsa-miR-222-3p	47	2.22E-08
	hsa-let-7a-5p		
Viral carcinogenesis	hsa-let-7a-5p	48	2.95E-07
Oocyte meiosis	hsa-let-7a-5p	35	4.06E-06
Protein processing in endoplasmic reticulum	hsa-miR-222-3p	37	4.08E-06
	has-miR-181b-5p		
Hepatitis B	hsa-let-7a-5p	42	2.87E-05
Adherents junction	hsa-let-7a-5p	28	8.11E-05
p53 signaling pathway	hsa-miR-222-3p	33	0.00013438
	has-miR-181b-5p		
	hsa-let-7a-5p		
Arrhythmogenic right ventricular cardiomyopathy (ARVC)	hsa-miR-222-3	16	0.00020225
	phsa-let-7a-5p		
Proteoglycans in cancer	hsa-let-7a-5p	58	0.00039485
	has-miR-181b-5p		
ECM-receptor interaction	hsa-let-7a-5p	15	0.00049296
Ubiquitin mediated proteolysis	hsa-miR-222-3p	53	0.00072422
	has-miR-181b-5p		
	hsa-let-7a-5p		
Endocytosis	hsa-let-7a-5p	45	0.0024329
Thyroid hormone signaling pathway	hsa-let-7a-5p	34	0.0026808
Bacterial invasion of epithelial cells	hsa-let-7a-5p	21	0.00606614
HTLV-I infection	hsa-miR-222-3p	64	0.01018435
	hsa-let-7a-5p		
TGF-beta signaling pathway	hsa-let-7a-5p	27	0.01158769
Pathways in cancer	hsa-let-7a-5p	79	0.01428958
Prostate cancer	has-miR-181b-5p	13	0.0242868
Transcriptional misregulation in cancer	hsa-let-7a-5p	40	0.02522058
Cell adhesion molecules (CAMs)	hsa-miR-222-3p	9	0.02693923
Chronic myeloid leukemia	hsa-let-7a-5p	20	0.02993537
Other types of O-glycan biosynthesis	hsa-let-7a-5p	7	0.03647756
Colorectal cancer	hsa-let-7a-5p	15	0.04248994
Renal cell carcinoma	has-miR-181b-5p	10	0.04614817

miRPath DIANA v3.0 software was used to perform KEGG analysis using TarBase database. Pathways with a *p* < 0.05 was considered significant after false discovery rate correction.

## Discussion

In this study we assessed the feasibility of using minimally invasive sEV- encapsulated miRNAs to discriminate IBC from non-IBC patients, as an alternative to invasive tissue biopsies. Our study showed that plasma sEV-encapsulated miR-181b-5p, miR-222-3p and let-7a-5p are potential biomarkers for IBC. Although, in last years, sEV-derived miRNAs showed significant relevance in various clinical and translational applications, the sEVs enrichment procedure could dramatically interfere with downstream applications [35, 36]. A major objective of our study was to optimize a procedure suitable for rapid and economical diagnosis and analysis of sEV-derived miRNAs. Thus, we first optimized the sEVs isolation protocol and compared different concentrations of PEG-based procedures to the gold standard ultracentrifugation and miRCURY exosome kit. We found that precipitating reagents did not only yield more sEVs (< 200nm) as examined by DLS, TEM, protein and RNA analyses, but also resulted in improved reproducibility. However, the miRCURY exosome kit unexpectedly showed broad size distribution in DLS with weak or no sEVs protein markers. This supports that commercial PEG-based kit may co-isolate larger EVs or huge amounts of sEV masking protein aggregates [14, 17]. DPEGT was optimized to specifically isolate sEVs through the combination of proteinase K and RNase A treatments to differentially centrifuged and ultra-filtrated plasma pooled samples. Based on DLS, RNA and protein patterns of isolated sEVs, the overall efficiency of DPEGT in retrieving sEVs was better than that of SPEGT and the miRCURY kit. This is in agreement with Yeing *et al*. [16], who stated that two step PEG-based precipitation was superior to single PEG, although they used different molecular weight of PEG to isolate sEVs from cell line conditioned media and without profiling of sEV-encapsulated miRNAs. Differences between 5, 8, 10, 12 and 15% of DPEGT were only evident through DLS not EV protein markers. Of note, sEV total RNA retrieved and miRNAs quantitative expression levels were significantly improved upon digesting enzymes treatment. Consistently, Moon *et al*. [37], have also highlighted the positive impact of proteinase k on improving different commercial kits purity for plasma EVs isolation. The monodispersed and paramount sEV yields obtained via DPEGT (8% + enzymatic treatment) made it the procedure of choice for subsequent miRNA profiling. Prior to downstream sEV-derived miRNAs analysis, the miRNA differential expression levels were measured, and no significant difference was detected between DPEGT and UC. Our study shows that high yields of sEVs could be efficiently enriched for miRNA profiling through an inexpensive PEG-based method. This is in consistent with Lv *et al*. [18], who used a PEG-based procedure for isolation of miRNAs from urinary exosomes. To test the feasibility of sEV-packaged miRNAs as a promising alternative for invasive tissue biopsies of IBC, we focused on three selected sEV-encapsulated miRNAs (miR-181b-5p, miR-222-3p and let-7a-5p), whose expression levels were detected in IBC tissues, as we recently reported [34].

The selected miRNAs are known for various oncogenic roles in breast cancer regulation as supported by the interaction networks analysis. Hub genes identified were mainly involved in regulation of chemokine signaling pathways, that trigger metastasis of IBC [38]. Based on KEGG pathways analysis, PI3K/AKT pathway that is remarkably activated in IBC [38], was shown to be regulated by these miRNAs. It has been shown that in gastrointestinal tumors miR-222 induces apoptosis through the activation of KIT and AKT pathway [39]. In ovarian cancer, miR-222 promotes chemotherapy resistance by targeting PTEN activating PI3K/AKT pathway [40]. Interestingly, miR181b sensitizes non-small cell lung cancer cells to chemotherapy by targeting directly TGFβR1/SMAD and indirectly PI3K/AKT pathway [41]. However, we did not find significant changes in expression levels of plasma sEV-packaged miR-181b-5p and miR-222-3p in non-IBC received neoadjuvant chemotherapy compared with untreated patients. Other PPI analysis has indicated that the tumor suppressor let-7a-5p is implicated in various pathways that regulate apoptosis, cancer stem cell differentiation and inhibits cells invasion and proliferation of cancer cells [42–44]. Interestingly, in line with our KEGG analysis and its role in cell cycle, it was reported that let-7a targeted the HMGA1 gene responsible for proliferation of breast cancer cells and direct modulation of miR-181b expression which subsequently downregulates CBX7, a protein responsible for the cell cycle at G1 phase [45, 46]. Multiple lines of evidence demonstrated that overexpressed miR-181 induces cancer progression [47–49]. It is noteworthy that IBC tumor overexpress the heparan sulfate proteoglycan syndecan-1 [50], aligning with KEGG pathway analysis, including proteoglycans in cancer. In late stage breast cancer tissues, transient expression of miR-181b is activated by STAT3, a transcription factor activated by IL-6 [51], which results eventually in upregulation of NF-kB and downregulation of let-7a [52, 53]. This is consistent with the upregulated levels of miR-181b-5p in IBC relative to stage-matched non-IBC. Given the hyperactivation of STAT3, NF-kB and upregulated IL-6 expression in IBC [54], we propose the same mechanism of regulation may be found in IBC. Remarkably, a significant upregulation of exosomal miR-181b than that free in plasma was proven in lung cancer patients, suggesting that sEV-derived miRNAs could efficiently mirror the expression pattern of carcinoma tissues [55]. In agreement with our results, Wang *et al*. [56], showed that the target genes of miR222-3p are involved in viral infection, proteoglycans in cancer and transcriptional misregulation in cancer. Consistently, upregulated exosomal miR-222 was reported to downregulate PDLIM2 tumor suppressor and to activate NF-κB, which results in lymph node metastasis in advanced breast cancer [57]. Among the pathways in cancer indicated by KEGG analysis, the upregulated miR-222 was involved in pivotal pathways, such as OSM, IL-6 and ERK/MAPK signaling pathways in breast cancer [58], known pathways to be activated in IBC [59, 60]. The overexpression of miR-222 was verified in IBC tissues [34, 61] and potentially used in management of breast malignancy. Noticeably, exosomal miR-222-3p can serve as a prognostic marker for different cancers [62, 63]. Interestingly, human papillomavirus E6 and E7 oncoproteins potentiated tumorigenesis possibly via exosomal miR-222 [64], supporting the strong association of viral infection and IBC [65, 66]. The KEGG analysis showed that hub genes, which are the targets of miR-181b-5p, miR-222-3p and let-7a-5p were associated also to synapsis. Interestingly, it was demonstrated that the miRNAs analyzed actively participate in the normal maturation and development of the central nervous system and in Alzheimer disease [67–71]. Although the mechanism and the important players have not been described, patients with IBC have a high incidence of developing metastasis to the brain associated with a poor prognosis. These sEV-derived miRNAs (miR-181b-5p, miR-222-3p and let-7a-5p) and their target genes could probably be involved in the metastasis of the IBC to the brain [72–74], possibly via induction of formation of pre-metastatic niches [75, 76]. Indeed, it has been described that some miRNAs, such as miR-181 and let-7 and other exosomal miRNAs participate in metastasis to the brain of breast cancer cells [77, 78]. Furthermore, plasma EV- derived let-7a-5p may emerge as a predictive marker for the response to neoadjuvant chemotherapy, as it is the only miRNA whose expression level was affected by neoadjuvant chemotherapy. In agreement, a study reported an association of let-7a-5p expression with drug sensitivity in breast cancer cell lines [79], however this needs further investigation for confirmation in clinical setting. Finally, the identified miRNAs are involved in SNARE interactions, that are crucial for the fusion of multivesicular bodies with the plasma membrane during the release of sEVs [80]. Taken altogether, these sEV-derived miRNAs and their enrichment in signaling pathways not only implies their critical involvement in IBC development and progression, but also indicate that they may serve as more precise and stable biomarkers than free plasma miRNAs for metastatic IBC diagnosis. Besides, the 3-miRNA expression profile in IBC vs non-stage- and stage-matched comparisons with non-IBC further strongly suggests their specific expression pattern in enriched plasma sEVs of IBC.

The usage of sEV-derived miRNAs as potential diagnostic biomarkers has been examined in different tumor entities. For example, miR-181-5p discriminated between adenocarcinoma and squamous cell carcinoma of early-stage lung cancer [81]. Serum exosomes-derived miR-222 is a diagnostic marker for patients with high- and low-grade gliomas [82]. However, to our knowledge, no studies introduced EV-derived miRNA panels for IBC diagnosis. But different studies have introduced EV-derived miRNAs to differentiate breast cancer and healthy subjects [23, 83]. EV-derived miR-21 and miR-1246 with AUC of 0.73 and miR-142-5p and miR-320a with AUC of 0.941 fulfilled such purpose [23, 83]. We explored the diagnostic potential of the plasma sEV-encapsulated miR-222-3p, let-7a-5p and miR-181b-5p that may help in differentiation of IBC from non-IBC, either individually or combined. The estimated correlations between the three miRNAs indicate their combinative value. Using ROC curve analysis, the maximum AUC were reported for let-7a-5p (AUC = 0.919) and for combined (miR-222-3p and let-7a-5p) (AUC = 0.973), with considerably high discriminatory values for each. No change was noticed between AUC values of the three recombined miRNAs and the panel of miR-222 and let-7a. Thus, the later emerges as a more efficient, precise, and cost-effective biomarker than individual miRNA for IBC detection.

Although not tested in this study, one possible limitation is that the proposed method may not be applied for mass spectrometric analysis studies unless simple purification steps are added, such as column chromatography, size-exclusion, or potassium chloride precipitation [84, 85]. However, the exosomal proteome of Hela cells was successfully profiled by a PEG-based method [16], suggesting the potential of our optimized PEG-method to discover exosomes contained specific diagnostic proteomic IBC biomarkers.

## Conclusions

In summary, this study introduced DPEGT procedure as a minimally invasive PEG-based procedure for the isolation of sEVs from plasma biopsy as an alternative approach for the painful invasive tissue biopsy. Among the isolated miRNAs, three miRNAs (miR-181b-5p, miR-222-3p, and let-7a-5p) showed dysregulated miRNA expression levels consistently with their expression in IBC tissue carcinoma. The combined miR-222-3p and let-7a-5p showed excelled diagnostic potency for discriminating IBC from non-IBC patients. Further studies are warranted to decipher the influence of cargo of isolated sEVs on behavior of the cells within tumor microenvironmental cells, namely tumor-associated-macrophages and- fibroblasts, as well as their therapeutic potential needs further investigation.

## Supporting information

S1 Fig Dynamic Light Scattering (DLS) for sEV size distribution. Comparison of sEV size distribution using different concentrations of (a) SPEGT and (b) DPEGT relative to ultracentrifugation and the miRCURY kit.
(TIF)

S2 Fig Gene stability of sEVs Reference Genes (RG). RefFinder assigns values of gene stability for each candidate reference gene based on their geometric mean. Comparable geometric means ranging from 1.19 to 2.28 for cel-miR-39, miR-16 and RNU6.
(TIF)

S3 Fig Expression of miR-181b-5p, miR-222-3p and let-7a-5p from enriched plasma sEVs in non-IBC patients with stage I, II and III. Relative expression levels of miR-181b-5p (a), miR-222-3p (b), and let-7a-5p (c) enriched in plasma sEVs of non-IBC patients with stage III (n = 10) and stage I or II (n = 24) by DPEGT. ns = not significant as determined by Student’s t-test.
(TIF)

S4 Fig Expression of miR-181b-5p, miR-222-3p and let-7a-5p from enriched plasma sEVs in neoadjuvant chemotherapy-treated and -untreated non-IBC patients. Relative expression levels of miR-181b-5p (a), miR-222-3p (b), and let-7a-5p (c) enriched in plasma sEVs of non-IBC treated (n = 12) and untreated patients (n = 19) by DPEGT. ** p < 0.01 and ns = not significant as determined by Student’s t-test.
(TIF)

S5 Fig The network of miR-181b-5p, miR-222-3p, and let-7a-5p targets protein interactors. String database output depicting functional and physical interactors of all targets from differentially regulated miRNAs.
(TIF)

S6 Fig Clustering and heat map of the KEGG enrichment pathways of miR-181b-5p, miR-222-3p, and let-7a-5p. miRPath DIANA v3.0 software was used to generate the figure.
(TIF)

S1 Table All the modules obtained from MCODE analysis in Cytoscape are showed. The most four significant modules (1–4), which contain the hub genes were selected. Later, these modules were submitted to String to find the KEGG pathways associated with these hub genes.
(XLSX)

S1 Raw images (ZIP)
