# Supplementary figures and images for: Small extracellular vesicle-encapsulated miR-181b-5p, miR-222-3p and let-7a-5p: Next generation plasma biopsy-based diagnostic biomarkers for inflammatory breast cancer
Source: PLoS One. 2021 Apr 26;16(4):e0250642. doi: 10.1371/journal.pone.0250642 (PMC8075236; doi:10.1371/journal.pone.0250642)

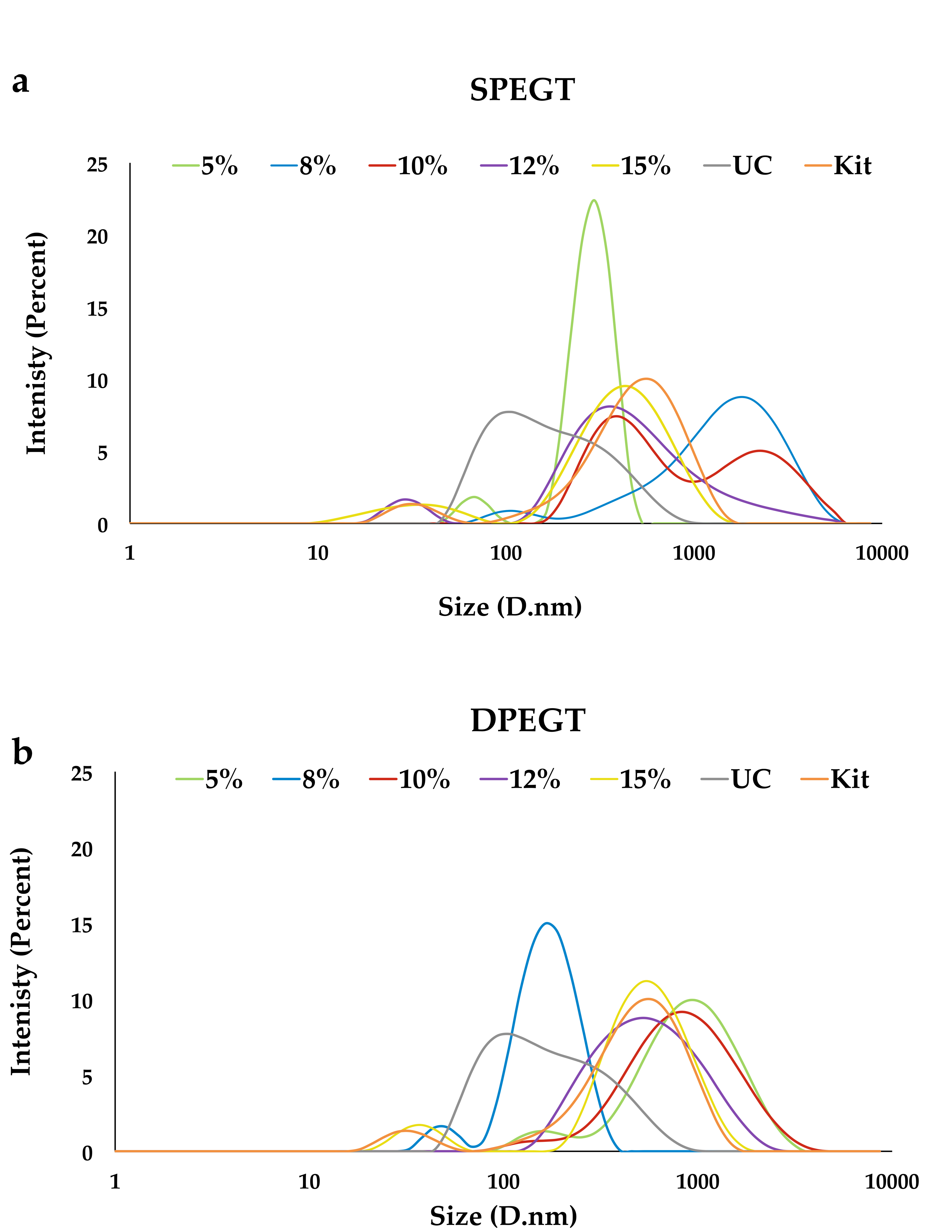

Supplement: S1 Fig — Comparison of sEV size distribution using different concentrations of (a) SPEGT and (b) DPEGT relative to ultracentrifugation and the miRCURY kit. (TIF) [file pone.0250642.s001.tif]

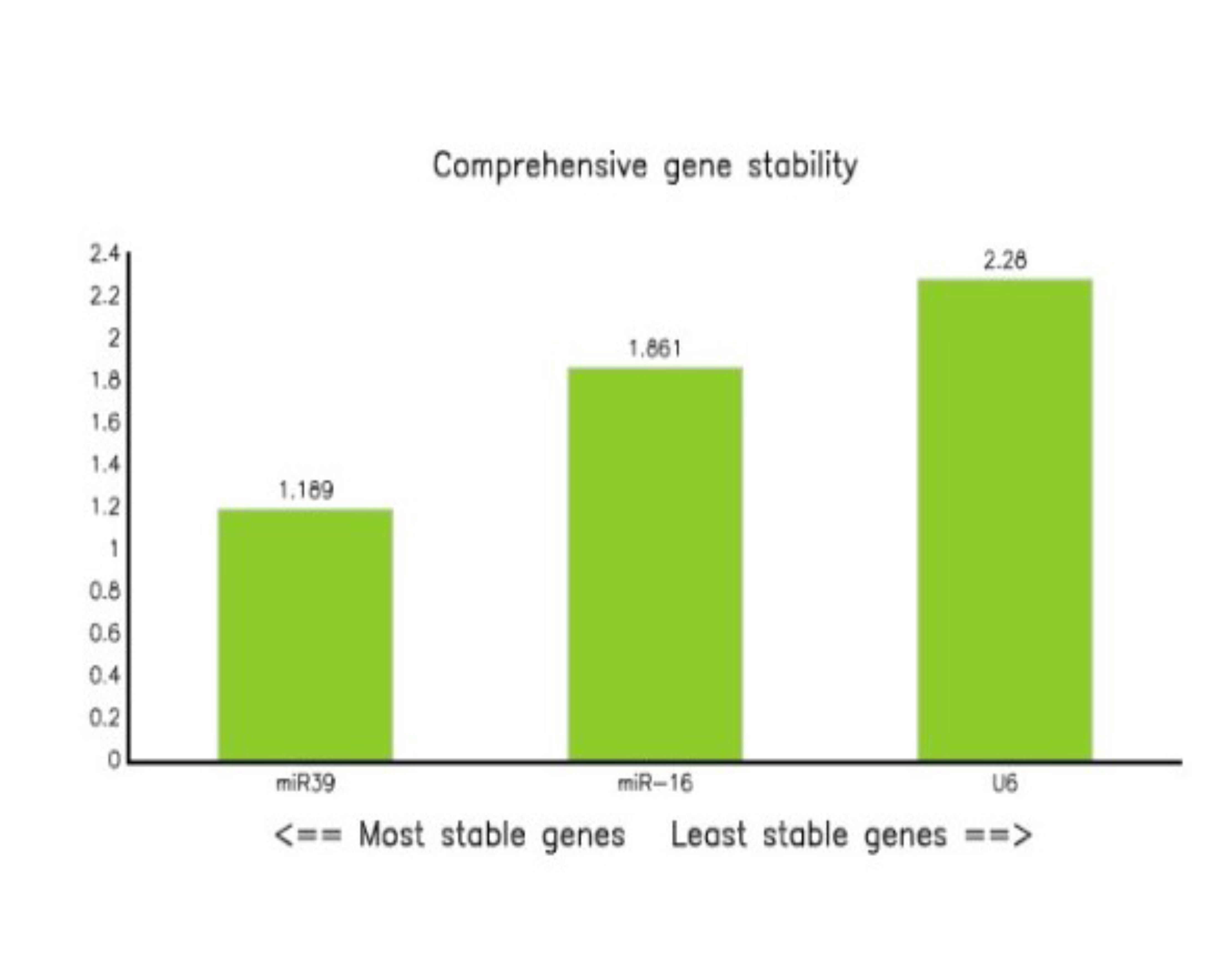

Supplement: S2 Fig — RefFinder assigns values of gene stability for each candidate reference gene based on their geometric mean. Comparable geometric means ranging from 1.19 to 2.28 for cel-miR-39, miR-16 and RNU6. (TIF) [file pone.0250642.s002.tif]

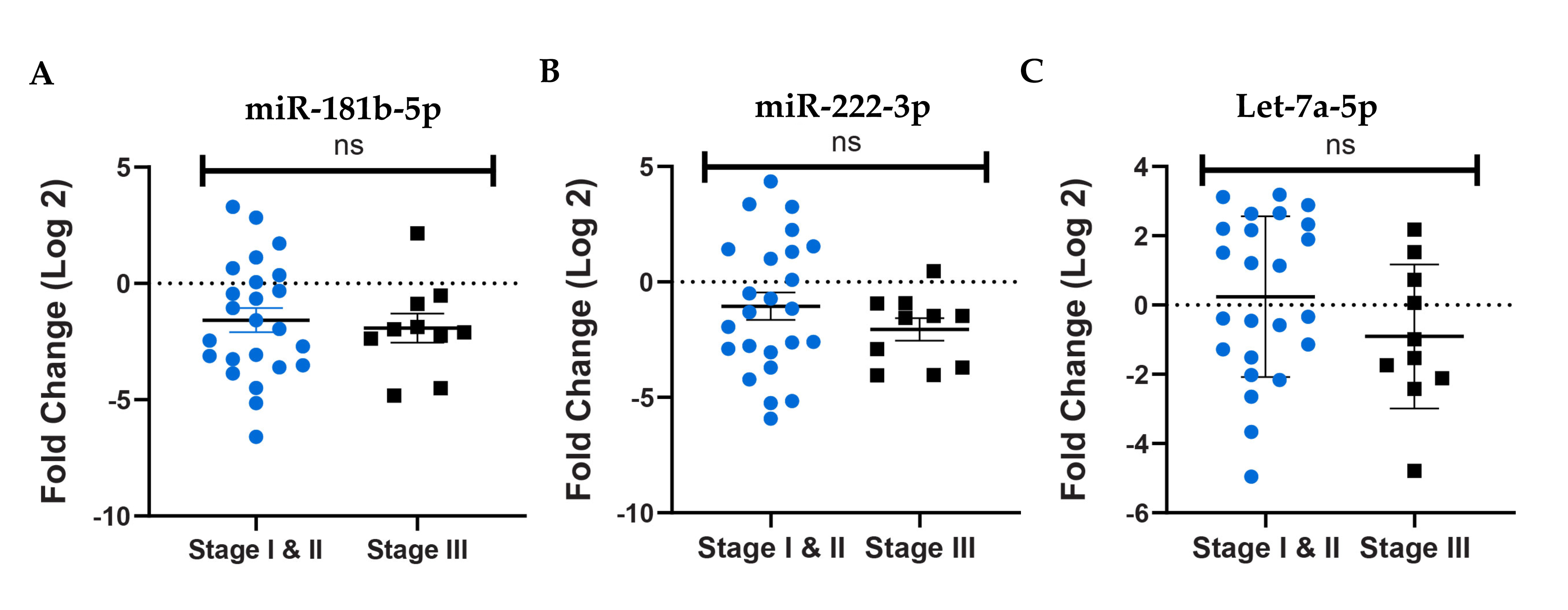

Supplement: S3 Fig — Relative expression levels of miR-181b-5p (a), miR-222-3p (b), and let-7a-5p (c) enriched in plasma sEVs of non-IBC patients with stage III (n = 10) and stage I or II (n = 24) by DPEGT. ns = not significant as determined by Student’s t-test. (TIF) [file pone.0250642.s003.tif]

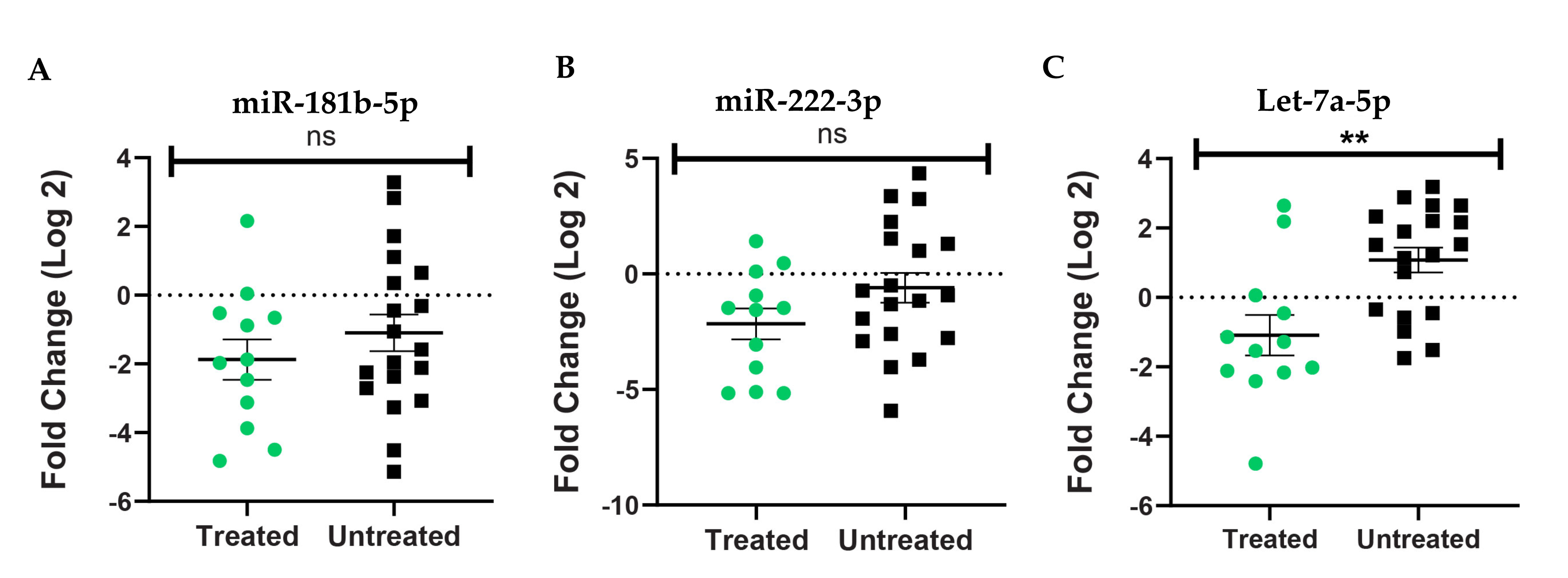

Supplement: S4 Fig — Relative expression levels of miR-181b-5p (a), miR-222-3p (b), and let-7a-5p (c) enriched in plasma sEVs of non-IBC treated (n = 12) and untreated patients (n = 19) by DPEGT. ** p < 0.01 and ns = not significant as determined by Student’s t-test. (TIF) [file pone.0250642.s004.tif]

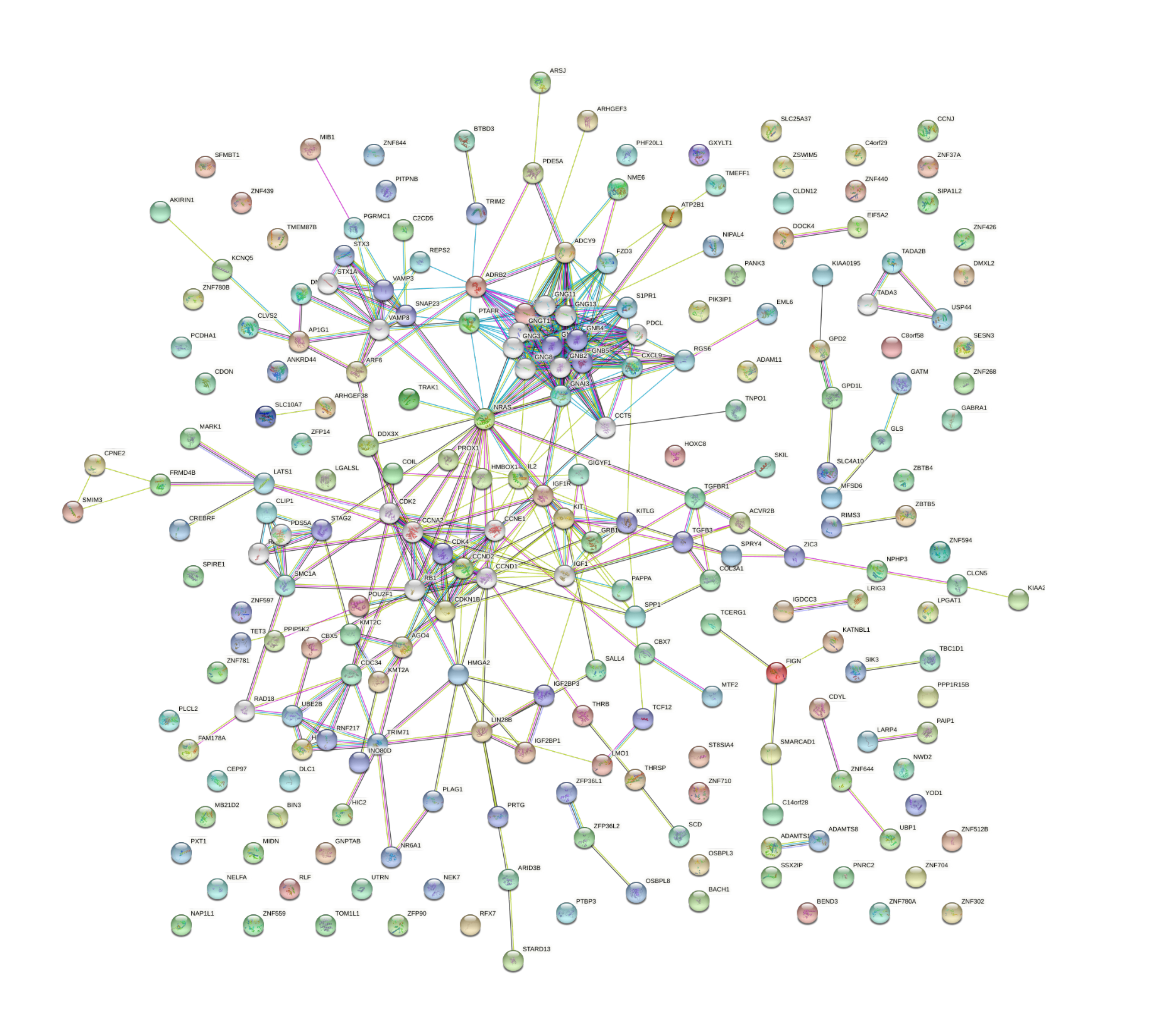

Supplement: S5 Fig — String database output depicting functional and physical interactors of all targets from differentially regulated miRNAs. (TIF) [file pone.0250642.s005.tif]

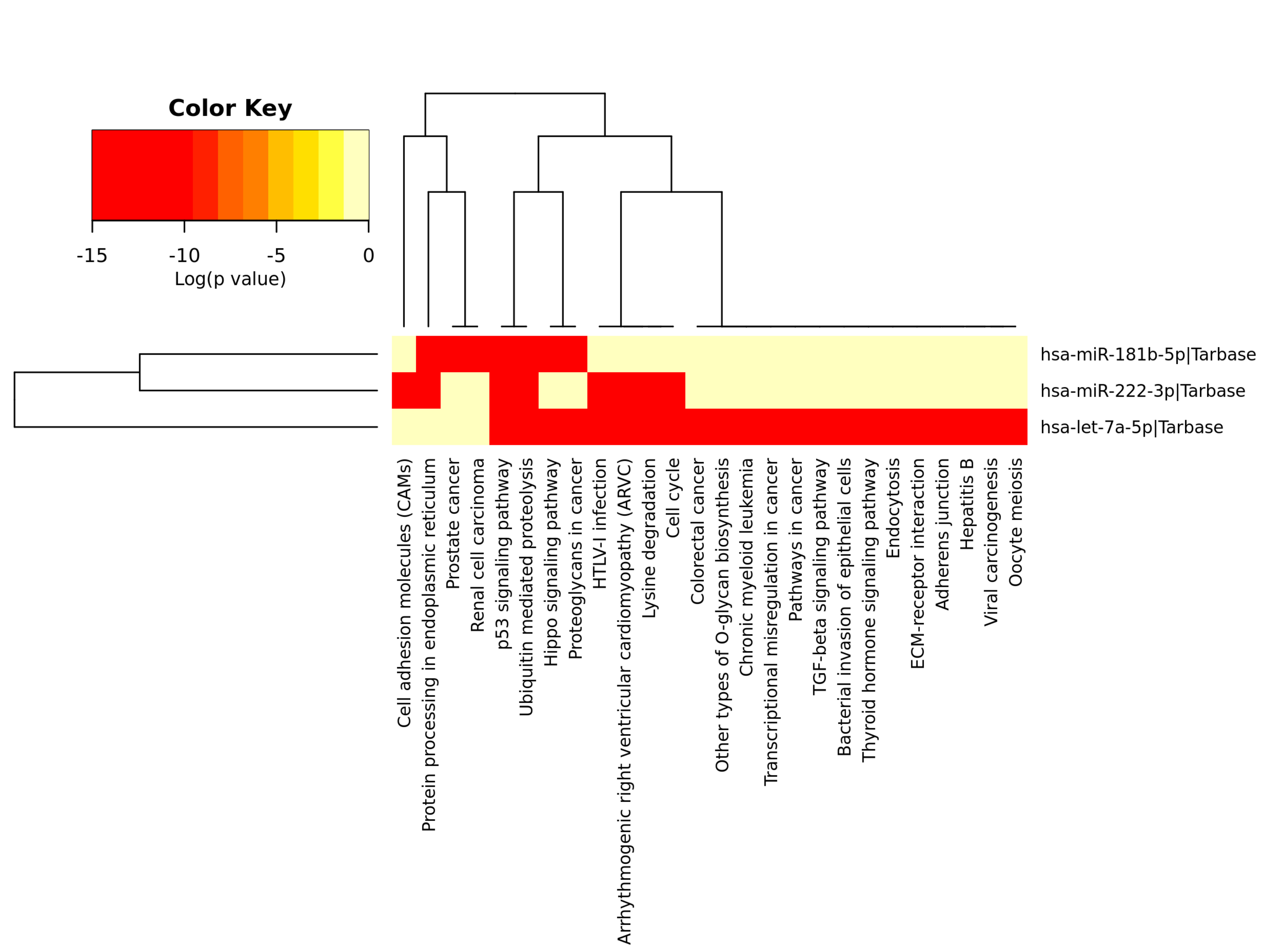

Supplement: S6 Fig — miRPath DIANA v3.0 software was used to generate the figure. (TIF) [file pone.0250642.s006.tif]

# ALIX

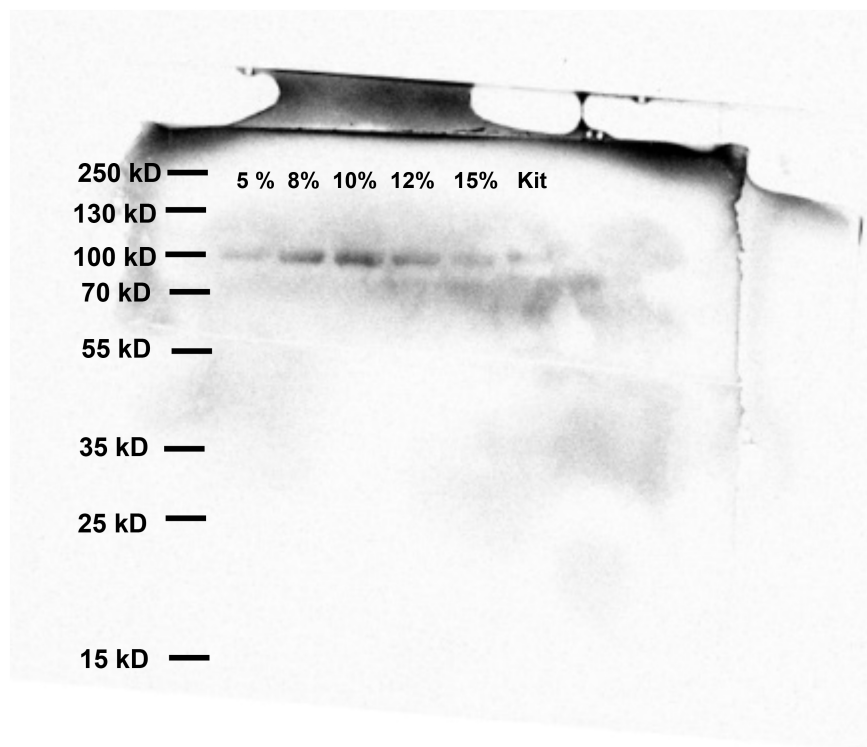

# HSP70

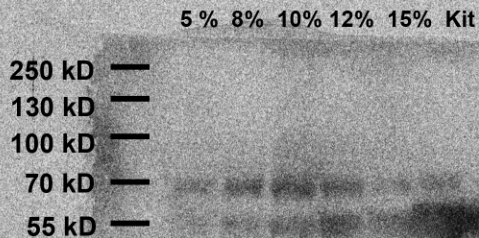

# CD63

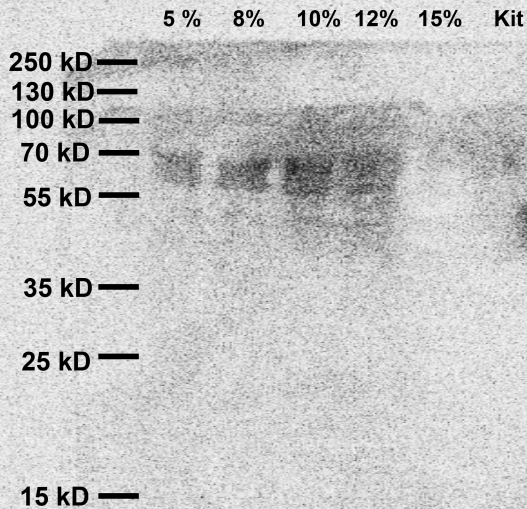

# GM130

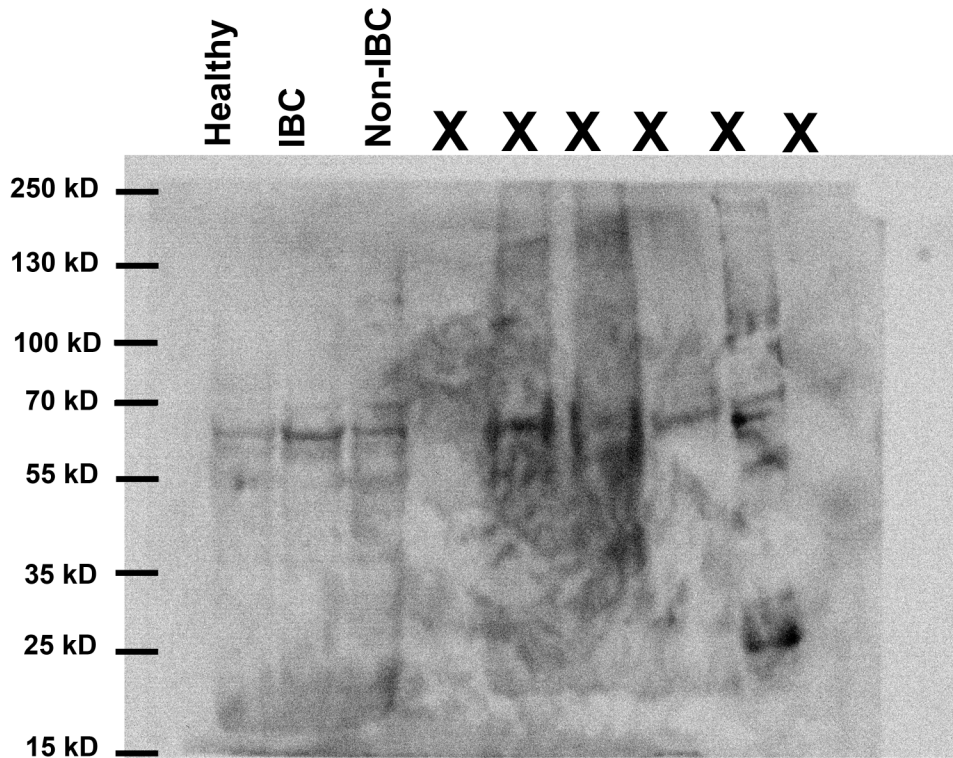

# CD63

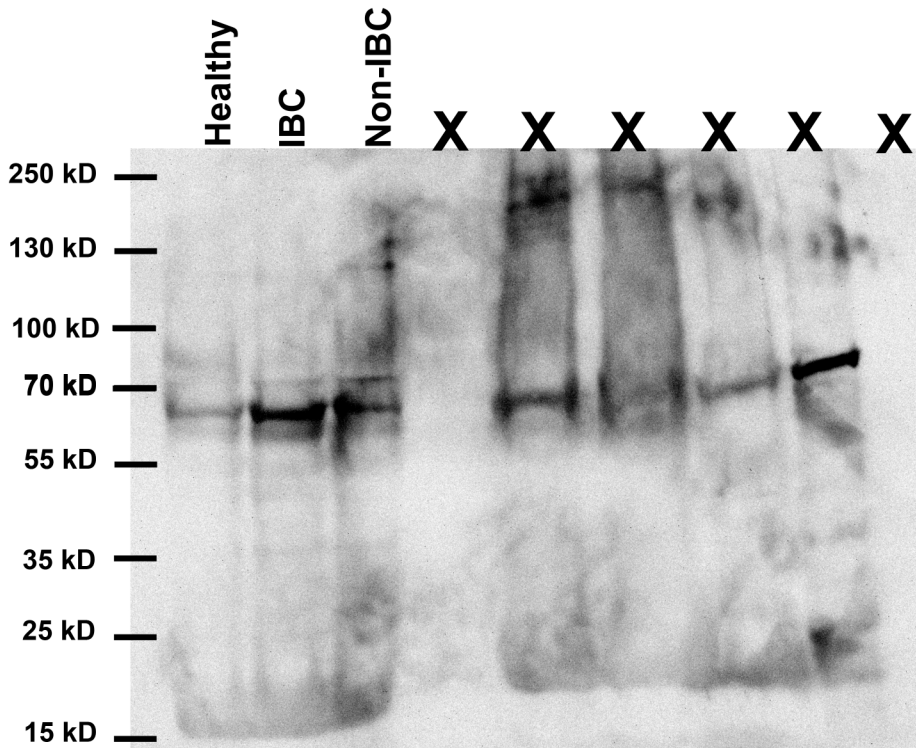

Supplement: S1 Raw images — (ZIP) [file pone.0250642.s008.zip › S1_raw_images.pdf]
